# Misato underlies visceral myopathy in *Drosophila*

**DOI:** 10.1038/s41598-017-17961-3

**Published:** 2017-12-18

**Authors:** Soohong Min, Woongchang Yoon, Hyunho Cho, Jongkyeong Chung

**Affiliations:** 1 National Creative Research Initiatives Center for Energy Homeostasis Regulation, Institute of Molecular Biology and Genetics, Seoul National University, 1 Gwanak-Ro, Gwanak-Gu, Seoul, 08826 Republic of Korea; 2 School of Biological Sciences, Seoul National University, 1 Gwanak-Ro, Gwanak-Gu, Seoul, 08826 Republic of Korea; 3 Harvard Medical School, Department of Cell Biology, 240 Longwood Avenue, Seeley-Mudd Building, Boston, MA 02115 USA

## Abstract

Genetic mechanisms for the pathogenesis of visceral myopathy (VM) have been rarely demonstrated. Here we report the visceral role of *misato* (*mst*) in *Drosophila* and its implications for the pathogenesis of VM. Depletion of *mst* using three independent RNAi lines expressed by a pan-muscular driver elicited characteristic symptoms of VM, such as abnormal dilation of intestinal tracts, reduced gut motility, feeding defects, and decreased life span. By contrast, exaggerated expression of *mst* reduced intestine diameters, but increased intestinal motilities along with thickened muscle fibers, demonstrating a critical role of *mst* in the visceral muscle. Mst expression was detected in the adult intestine with its prominent localization to actin filaments and was required for maintenance of intestinal tubulin and actomyosin structures. Consistent with the subcellular localization of Mst, the intestinal defects induced by *mst* depletion were dramatically rescued by exogenous expression of an actin member. Upon ageing the intestinal defects were deteriorative with marked increase of apoptotic responses in the visceral muscle. Taken together, we propose the impairment of actomyosin structures induced by *mst* depletion in the visceral muscle as a pathogenic mechanism for VM.

## Introduction

Animals possess a segregated layer of musculatures called visceral muscle on the surface of gastrointestinal tract with distinct morphology and functions in comparison to the other types of musculatures such as skeletal and cardiac muscles^1,2^. In particular, the visceral muscle is comprised of the circular and longitudinal muscles producing peristalsis to facilitate mechanical digestion and transportation of ingested food along intestinal tract^3,4^. Besides these digestion-related functions, the visceral muscle serves as a niche for intestinal stem cells to differentiate into various intestinal cells by secreting a blend of proliferative factors^5^. Due to these highly specialized functions, abnormalities in the visceral muscle are often associated with a spectrum of intestinal diseases in humans^6^. Particularly, degeneration of the visceral muscle along with fibrosis is the hallmark of visceral myopathy (VM) accompanying intestinal dilation and obstruction, deficient bowel movement, abdominal pain, and malnutrition^7^. Although VM is a rare disease, pathogenic symptoms are severe and often familial^8,9^. A member of actins specific to smooth muscles was previously suggested as a causative factor for VM through genetic studies and genome-wide sequencing^10–12^, however, whether intervention of a specific gene *in vivo* is linked to VM has remained elusive.

The *Drosophila* intestine provides an excellent model system to investigate the genetic and pathogenic mechanism underlying VM. First of all, the *Drosophila* intestine preserves most aspects of the vertebrate system, including visceral muscles and epithelial intestinal cells specialized for absorbing nutrients and secreting hormonal factors^13^. Like vertebrates, the *Drosophila* visceral muscle consists of inner and outer layers of circular and longitudinal muscles^14^. Extensive studies using *Drosophila* have been performed to reveal how visceral muscle contributes to the cellular homeostasis in the intestine including regulation of intestinal stem cells by visceral muscle-derived factors such as Wingless/Wnt and epidermal growth factor^15,16^. Besides these anatomical conservations, a plenty of genetic tools are available^17^. For example, visceral muscle-specific driver lines, and gene silencers and activators allow one to easily intervene or potentiate expression of the genes involved in the function of the visceral muscle by driving exogenous genes and RNAi in *Drosophila*. Furthermore, alterations in the structure of the visceral muscle by genetic manipulations can be thoroughly traced using diverse genetic reporters *in vivo*.

*Misato* (*mst*) encodes a protein that is highly conserved among animal species and that retains a mixture of protein motifs found in tubulins and myosins^18^. In *Drosophila*, *mst* null mutation was shown to elicit larval lethality associated with abnormal chromosomal segregation during cell division^19^. Mst was also shown to regulate the formation of mitotic spindles during mitosis by interacting with the TCP-1 tubulin chaperone complex^20^. However, the protein encoded by *mitochondrial distribution and morphology regulator* (*MSTO1*), the orthologue of *mst* in human, was shown to localize to mitochondria to regulate subcellular distribution of mitochondria and their morphology^21^. Studies have implicated that MSTO1 interacts with some factors including caspases, transcriptional components and actin-related proteins involved in intestinal cancer and VM^22,23^. In addition, an investigation on patients with inflammatory bowel disease revealed an SNP on the locus 1q22 containing *MSTO1* ^24^.

Through a genetic screen using a collection of RNAi libraries, we found that depletion of *mst* in muscle tissues caused the VM-like traits including intestinal dilation, reduced gut motility, defective food intake, and shortened life span. Our data consistently supported that *mst* was required for visceral muscle maintenance via controlling actomyosin structures. These results led us to propose that the intestinal abnormalities caused by *mst* depletion in the *Drosophila* visceral muscle are a pathologic model for VM.

## Materials and Methods

### Fly food and stocks

All the fly stocks were maintained on a standard cornmeal agar food containing dextrose 1,260 g, cornmeal 635 g, agar 91 g, yeast 900 g, propionic acid 84 ml, and tegosept 132 ml in 18 L of food manufactured by KAIST *Drosophila* Library Facility in Korea. Fly driver lines used in this study: *Act5c-GAL4* [Bloomington *Drosophila* Stock Center (BDSC), IN, ID: 25374], *Cg-GAL4* (BDSC: 7011), *nSyb-GAL80* (Dr. Julie Simpson, Janelia Farm, VA), *UAS-mCD8GFP* (BDSC: 5137], *Tub-GAL80* ^*ts*^ (Dr. Ron Davis, Scripps Research Institute, CA), *how-GAL4* (Dr. Won-Jae Lee, Seoul National University, Korea), *5053A-GAL4* (Dr. Won-Jae Lee, Seoul National University, Korea), *Myo1A-GAL4* (Kyoto Stock Center, Japan, ID: 112001), and *mef2-GAL4* drivers (Bour *et al*., 1995). *mst* RNAi lines: 1424R-1 (National institute of Genetics, Japan) (BDSC: 29601) and 110422 [Vienna Drosophila Resource Center (VDRC), Austria]. *mst* transgenic lines: *UAS-mst* (FlyORF F001471) and *UAS-mstGFP* (Dr. Silvia Bonaccorsi, University of Rome, Italy). Apoptosis-related lines: *UAS-p35* (BDSC: 5072), *Dronc RNAi (1)* (BDSC: 32963), *Dronc RNAi (2)* (VDRC: 100424), *Dronc RNAi (3)* (VDRC: 23035), *Dredd RNAi* (BDSC: 34070), and *nej RNAi* (BDSC: 31728). Actin-related RNAi lines: *Act5C RNAi* (BDSC: 42651), *Act42A RNAi* (BDSC: 50625), *Act57B RNAi* (BDSC: 31551), *Act79B RNAi* (BDSC: 36857), *Act87E RNAi* (BDSC: 42652), *Act88F RNAi* (BDSC: 60347), *Arp53D RNAi* (BDSC: 44580), *Actinin RNAi* (BDSC: 34874), *Actinin 3 RNAi* (BDSC: 26737), and *Bent RNAi* (BDSC: 31545 and 31546). Actin::GFP lines: *Act5C::GFP* (BDSC: 9258), *Act42A::GFP* (BDSC: 9251), *Act57B::GFP* (BDSC: 9256), *Act79B::GFP* (BDSC: 9248), *Act87E::GFP* (BDSC: 9249), and *Act88F::GFP* (BDSC: 9254).

### Generation of transgenic flies

DNA sequences in *mst* to be deleted for tubulin-like and myosin-like motifs were determined by the previous report^18^. Primers used for generation of Mst Δ1, 2 and 3 lines: Mst Δ1: Forward 5′-GCCGAATTCATGCAGCAGGAGGCCAACTTTAG-3′, Reverse 5′-GGCCTCGAGTAGATACTGCGAGTCTGC-3′. Mst Δ2: Forward 1 5′-GCCGAATTC ATGGACTATACACGTG-3′, Reverse 1 5′-GGCGCGGCCGCCCTCATCACGCTGCACAAA-3′, Forward 2 5′-GCCGCGGCCGCAAGAACTATCAGCTGGCAGC-3′, Reverse 2 5′-GGCCTCGAG TAGATACTGCGAGTCTGC-3′. Mst Δ3: Forward 1 5′-GCCGAATTCATGGACTATACACGTG-3′, Reverse 1 5′-GGCGCGGCCGCCGCAGAACTCCTCGTTGAAG-3′, Forward 2 5′-GCCGCGGCCGCGAGCACCTCAACGATGAGTAC-3′, Reverse 2 5′-GGCCTCGAGTAGATACTGCGAGTCTGC-3′. The PCR products using these primers were digested by restriction enzymes of EcoRI, XhoI and NotI, and inserted into pUAST-HA vector. The full sequence of Mst Δ2 and Mst Δ3 was obtained to confirm mutations. The pUAST-HA vectors containing cDNAs encoding Mst Δ1, Mst Δ2 and Mst Δ3 were midi-prepped and injected into fly embryos to generate transgenic flies.

### Feeding and proboscis extension response (PER) assays

The assays were performed as previously described using the flies with indicated genotypes for each experiment^25^.

### Gut contraction measurement

A fly aged for 5 days was immobilized on a silicon plate filled with 1 × PBS using two insect pins fixed across its neck. The plate and forceps for dissection should be free of fixatives such as paraformaldehyde (PFA) prior to dissection. The whole gut was dissected out of the body in short time by removing the remaining tissues including cuticle, skeletal muscle, fat body, and reproductive organs with extreme care not to damage the intestine. After dissection, the frequency of gut contraction for a minute was counted by visual inspection.

### Gut diameter

The fixed intestine stained with phalloidin was imaged under a confocal microscope (LSM710, Carl Zeiss, Germany), and the Z-stack projection image of the intestine was obtained. The diameter of an intestine was determined by measuring at least three different locations in the intestine using ZEN 2009 Light edition software (Carl Zeiss, Germany).

### Fecal spots

Three flies aged for 5 days were housed in a vial containing green dyed food for 24 hours. On the next day, the fecal spots on a white plug from the vial were counted. The total number of the spots was divided by three to obtain the number for a single fly.

### Life span

Thirty to forty virgin females were crossed with more than five male flies in a fly bottle. The newly-born F2 progenies were collected and sorted into a vial containing the regular fly food with equal sexual ratio. The number of dead flies was counted every day until all the flies in experimental groups were dead and the survival curve was drawn using Prism 5 software (Graphpad, CA).

### Immunohistochemistry on fly intestine and other tissues

Flies aged for 3–5 days were dissected in 1 × PBS for the intestine, thorax and brain. The dissected tissues were then fixed with 4% PFA for 20 minutes and washed with 0.1% PBST 2 times for 5 minutes. The tissue was permeabilized for 10 minutes with 0.5% PBST on a nutator. The permeabilized tissue was washed with 0.1% PBST 3 times for 5 minutes and incubated with 3% bovine serum albumin in 0.1% PBST for 30 minutes at room temperature (RT). Primary antibodies including anti-mouse Mst antibody (Santacruz, sc-13568, 1:200), anti-mouse tubulin antibody (Abcam, ab6046, 1:200), and anti-mouse myosin heavy chain antibody (DSHB, ALD-58, 1:100) were treated with the noted ratios for overnight at 4 °C. On the next day, the primary antibody solution was discarded and the tissues were washed by nutating in 0.1% PBST 3 times for 5 minutes and the following solutions were treated for 2 hours at RT in dark: anti-mouse Alexa fluor 647 (1:200), anti-phalloidin antibodies conjugated with FITC and TRITC (1:200), and Hoechst (1:400). The tissues were washed in 0.1% PBST 3 times, and then washed in PBS once for 5 minutes and mounted on a slide glass with SlowFade mounting solution (Invitrogen, CA, ID: S36936).

### Immunoblotting using fly intestine

Prior to blotting, the intestines from fifteen flies aged 5–8 days were prepared in Triton X-100 lysis buffer (20 mM Tris-Cl pH 7.5, 100 mM NaCl, 1 mM Na_2_VO_4,_ 1 mM EDTA, 1 mM EGTA, 50 mM β-glycerolphosphate, 50 mM NaF, 1% Trition X-100 (v/v), 1 mM PMSF, 10 g/mL leupeptin, and 1 g/mL pepstatin A). The intestines were homogenized, and lysates were subjected to SDS-PAGE analysis according to standard procedure. The immunoblots were developed and visualized using LAS-4000 (Fuji Film, Japan).

### Quantitative real time PCR

To quantify expression of *mst*, we isolated total RNA from intestines and thorax muscles from fifteen *mef2-GAL4* fly stocks using TRIzol (Invitrogen, CA). To knockdown *mst* expression, we combined each UAS-*mst* RNAi transgene with *mef2-GAL4* line. To quantify the efficacy of RNAi in the intestine, we extracted total RNA from fifteen adult flies. Total RNA (1 μg) from each sample was used as a template for reverse transcription using M-MLV Reverse Transcriptase (Promega, WI). cDNA preparation was subjected to real-time quantitative PCR (Bio-Rad CFX96 Real-Time PCR detection system, CA) according to the protocol for SYBR Green Mix (Enzynomics, Korea, ID: RT500). The primers used for real-time quantitative PCR: Forward 5′-CTTCCACGTGCTGTTCGACA-3′, Reverse 5′-CATCAAAGCCTGCTCGCTGA-3′. In each reaction, we normalized expression of *mst* transcripts to *rp49*.

### TUNEL assay

Flies aged for 3–5 days were dissected in 1 × PBS to obtain intestines. The dissected intestines were fixed in 4% PFA for 20 minutes at RT. The fixed intestines were washed with 0.5% PBST twice for 10 minutes. 0.1 M sodium citrate in 0.1% PBST was added and the sample was incubated at 65 °C for 30 minutes. The sample was washed with PBS twice for 10 minutes. As a positive control, DNase solution (1.5 μl of DNase, 3 μl of 10 mg/ml BSA, 1.5 μl of 1 M Tris buffer with pH7.5, and 24 μl of H_2_O) was added into a control sample. For TUNEL reaction, experimental samples were incubated in 2 μl of TUNEL enzyme (TMR red, 12156792910, Roche, Swiss) and 18 μl of buffer at 37 °C for 2 hours. After the reaction period, the samples were washed with 0.1% PBST for 10 minutes, and additional staining markers such as phalloidin-TRITC and Hoechst were added and incubated for 1 hour at RT. The staining markers were removed, and the samples were washed with 0.1% PBST three times for 10 minutes.

### Phylogenetic analysis

The *mst* orthologues were extracted from HomoloGene in the NCBI and Pan-taxonomic compara in the Ensembl. We aligned known *mst* paralogue protein sequences from human to fruit fly and analyzed conserved sequences related to the function of *mst*. The *mst* orthologue protein sequences for *Homo sapiens*, *Bos Taurus*, *Mus musculus*, *Rattus norvegicus*, *Silurana tropicalis*, *Danio rerio*, and *Drosophila melanogaster* were downloaded from Uniprot. The Clustal Omega was used to generate sequence alignments. Aligned sequences were visualized and annotated with the Jalview.

### Protein-protein interaction network analysis for *mst* and VM-related genes

Annotated data and associated genes linked to intestinal pseudo-obstruction-related diseases were extracted from Clinvar, MalaCards, OMIM, Orphanet, and UniProtKB/Swiss-Prot databases. Protein-protein interaction network was constructed with intestinal pseudo-obstruction association genes and known Mst protein-protein interaction genes extracted from the interactome databases, BioGRID and IntAct. We analyzed a high-ranked gene set using the shortest path from *mst* to other pathogenic genes. The Cytoscape 3 was used to construct, visualize, and analyze the network.

## Results

**Figure 1:**
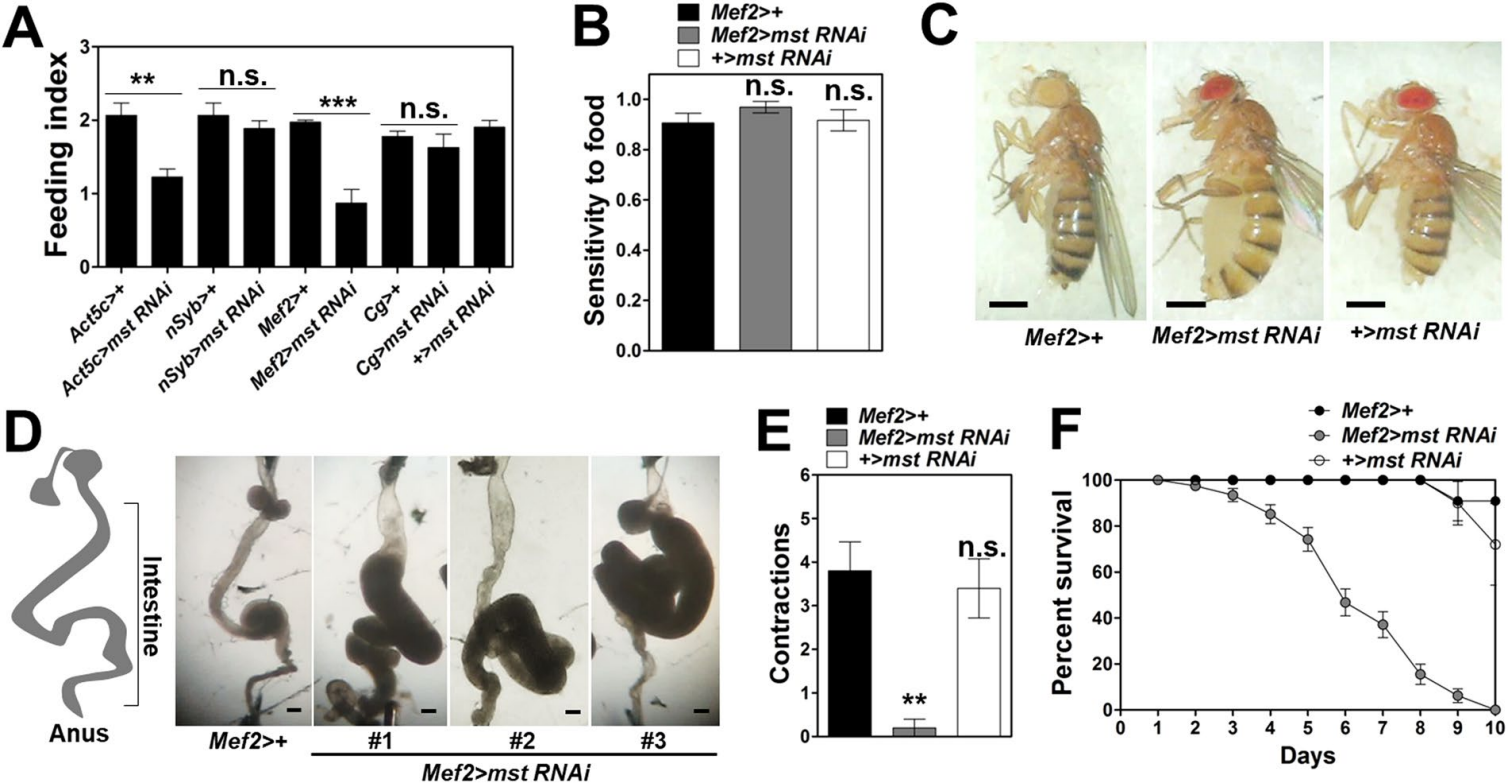
Pan-muscular depletion of *mst* elicits feeding defects, intestinal dilation, reduced gut motility and shortened life span. (**A**) Comparisons of the level of food intake by the flies with indicated genotypes, N = 3, **p < 0.005; ***p < 0.0005; n.s. not significant by unpaired t-test. (**B**) Comparison of the frequency of proboscis extension responses (PER) to sucrose by the flies with indicated genotypes, N = 12. n.s. not significant by ANOVA Dunnett’s multiple comparison test. (**C**) Whole body images of the flies with indicated genotypes. Scale bars: 0.5 mm. (**D**) Schematic of the intestinal tract of the fly and bright field image of the flies with indicated genotypes. Scale bars: 200 μm. (**E**) Comparison of the frequency of the gut contractions per 1 minute with indicated genotypes. N = 5. **p < 0.005 by ANOVA Dunnett’s multiple comparison test. (**F**) Comparison of the survivorship of the flies with indicated genotypes. N = 3–5. Statistical significance was analyzed by Log-rank (Mantel-Cox) test. p < 0.0001.

To isolate genes that are critical for food intake, we performed a genetic screen using a collection of ~250 UAS-RNAi lines (Fig. S1A). These RNAi lines were against a list of genes that are presumed to be expressed in the outer membrane of mitochondria. Considering that this organelle serves as a key energy producing compartment in the cell, we hypothesized that a particular group of mitochondrial genes monitors internal energy level in a whole organism and ultimately orchestrates food intake. For example, mitofusins that control mitochondrial dynamics are reported to regulate food intake via their roles in the subset of hypothalamic neurons that dictate food intake in response to feeding and fasting conditions^26^. By crossing this mitochondrial RNAi collection to a whole body GAL4 line, *Act5C-GAL4*, we obtained progenies where individual genes were depleted in the whole body. The progenies were then examined in a high throughput feeding assay which allows us to examine a gene’s function in food intake. However, many of the RNAi lines crossed to the ubiquitous GAL4 driver caused lethality and thus they were skipped from our food intake screen. After all, we identified an RNAi line against *misato* (*mst*) that showed a marked decrease in food intake when crossed to *Act5C-GAL4* (Fig. S1A, arrow). Previously, it was reported that a null mutation of *mst* caused larval lethality^20^ and depletion of *mst* using other whole body GAL4 drivers such as *tubulin-GAL4* (*tub-GAL4*) and *daughterless-GAL4* (*da-GAL4*) elicited developmental lethality (data not shown). Interestingly, depletion of *mst* in neurons and fat body did not show any sign of defects in food intake (Fig. 1A), whereas the flies with muscle-specific knockdown of *mst* by *mst* RNAi expression driven under the control of *mef2-GAL4*, a pan-muscular driver, (henceforth, *mef2* > *mst RNAi*) exhibited significantly decreased food intake (Fig. 1A) without affecting the sensitivity to food (Fig. 1B). Notably, *mef2* > *mst RNAi* flies exhibited abnormal distention of their belly even though the flies ate less compared to controls (Fig. 1C), suggesting the possible correlation of the abdominal phenotype with the feeding defect. To examine whether the distended abdomen is owing to abnormalities in the size of internal organs including the intestine and the reproductive organs, we dissected and examined *mef2* > ms*t RNAi* flies. Remarkably, we observed severe dilation of intestinal tracts from all three different *mst* RNAi lines including *mst* RNAi #1 used in the food intake screen (Fig. 1D). To examine whether the intestinal phenotype is due to any developmental defects by *mst* depletion, we restricted the *mst* knockdown in the adulthood using the temperature sensitive GAL80 combined with *mef2-GAL4* (*mef2* ^*ts*^ >+) to induce expression of *mst* RNAi in the muscle (*mef2* ^*ts*^ > mst *RNAi*) only after birth at the non-permissive temperature (30 °C). We observed that *mst* knockdown in the adult still produced dilation of the intestine (Fig. S1B). Along with this intestinal phenotype, we also observed that *mef2* > m*st RNAi* flies showed less frequent contraction of the intestine, shortened life span, increased gut permeability, and elevated infection compared to controls (Figs 1E and F, S1C, and D, respectively), indicating *mst* depletion affected not only intestinal morphology but also intestinal homeostasis.

We raised a possibility that these intestinal phenotypes could be due to the failure of food digestion. Thus, we examined the possibility by feeding *mef2* > *mst RNAi* flies with agar-containing food and liquid food. Supporting the possibility, we observed that *mef2* > *mst RNAi* flies reared on liquid food exhibited significantly ameliorated survivorship compared to those grown on agar-containing food (Fig. S1E). These results indicate that *mst* plays critical roles in the visceral muscle to maintain intestinal integrity and motility for digestion.

Based on the result that *mef2-GAL4* driver induced gene expression not only in muscles but also in significant proportions of the central nerve system (Fig. S2A), we sought to examine which tissue is critical for the intestinal phenotypes. We paid first attention to the necessity of neuronal tissues for the phenotypes by inclusion of *nSyb-GAL80* in *mef2* > *mst RNAi* flies. The pan-neuronal expression of GAL80 specifically blocks the transcriptional activity of GAL4 to drive *mst* RNAi expression in neuronal tissues. We observed that neuronal blockade of *mst* RNAi expression in *mef2* > *mst RNAi* flies still elicited dilation of the intestine similarly as appeared in *mef2* > *mst RNAi* flies (Fig. S2B), indicating that neuronal tissues are dispensable for the intestinal phenotypes. On the other hand, skeletal muscle-specific knockdown of *mst* using *mhc-GAL4* ^27^ crossed to *mst* RNAi did not elicit the intestinal dilation (Fig. S2C). Since *mef2-GAL4* induced gene expression in skeletal muscles (Fig. S2A), we wondered whether *mef2* > *mst RNAi* flies show any defects in skeletal muscle morphology and locomotive activity. However, these flies exhibited fairly normal skeletal muscle structures and locomotive activity as seen in control flies (Fig. S2D and E). Together, these results suggest that *mst* depletion specifically affects visceral muscles.

**Figure 2:**
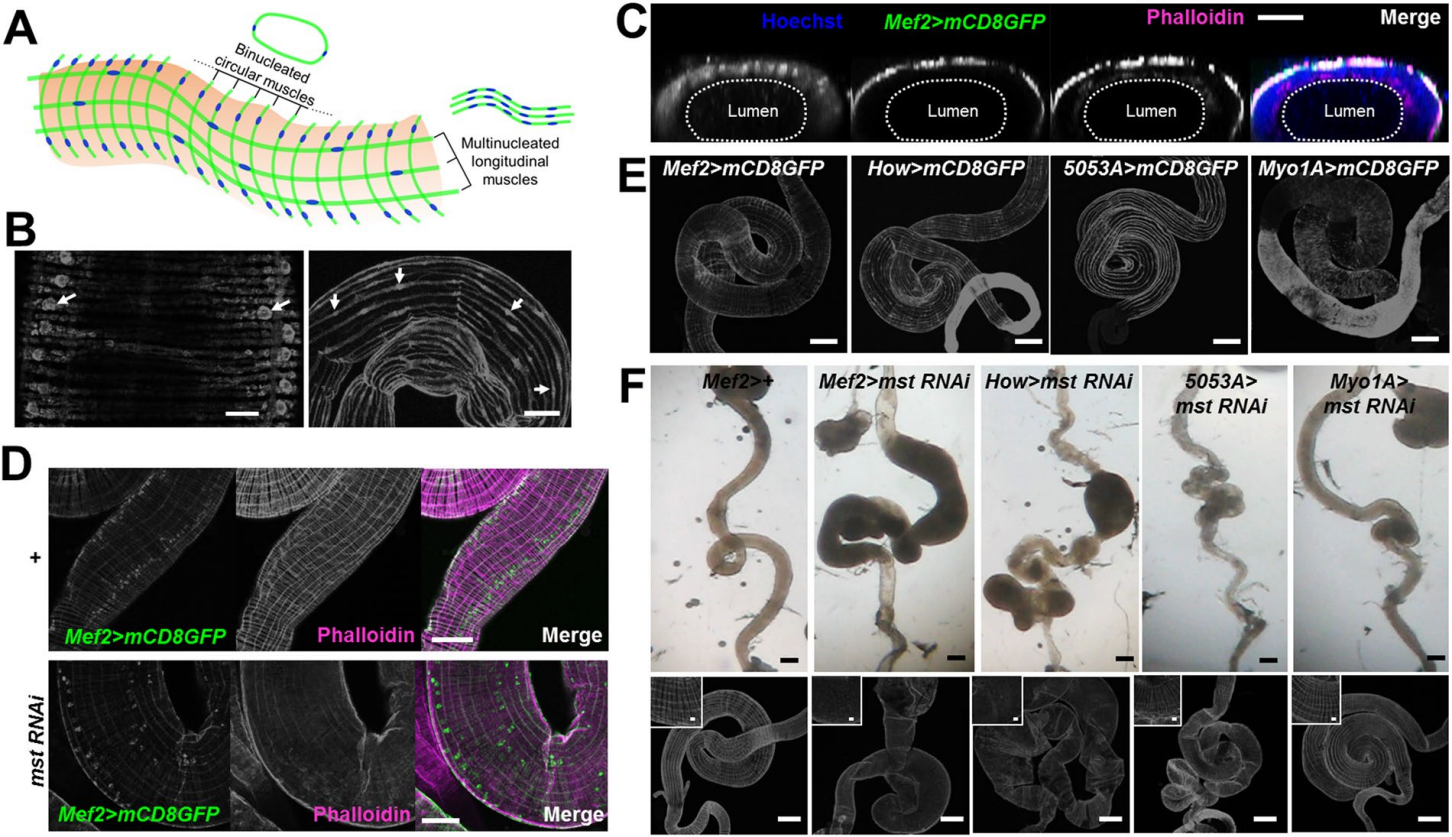
Visceral muscle is critical locus for the *mst* RNAi-induced phenotypes. (**A**) Schematic drawing of *Drosophila* visceral muscle comprised of the binucleated circular and the multinucleated longitudinal muscles. (**B**) Confocal images of the binucleated circular and the multinucleated longitudinal muscles expressing mCD8GFP with arrows indicating the nucleus in a muscle cell. Scale bars: 20 and 100 μm. (**C**) Vertical section of the confocal image of the intestine expressing *mef2* > *mCD8GFP* stained with Hoechst and phalloidin. Note that *mef2-GAL4* driven GFP was localized only at the exterior layer of visceral muscle. Scale bar: 50 μm. (**D**) Confocal images of the visceral muscles expressing *mef2* > *mCD8GFP* with indicated genotypes stained with phalloidin. Scale bars: 100 μm. (**E**) Confocal images of the intestine expressing mCD8GFP driven by *mef2-*, *how-*, *5053A-* and *myo1A-GAL4*. Scale bars: 200 μm. (**F**) Bright field and confocal images of the intestine with indicated genotypes. Insets are the zoomed-in images of the intestine stained with phalloidin. Scale bars: 200 μm.

The visceral muscle is known to be comprised of the binucleated circular muscle and the multinucleated longitudinal muscle along the intestinal tract in *Drosophila* (Fig. 2A and B)^28,29^. To visualize which part of the *Drosophila* visceral muscle is labeled by *mef2-GAL4*, we co-stained the intestine expressing mCD8GFP driven by *mef2-GAL4* with phalloidin, a potent muscle marker reactive to F-actin^30^. Phalloidin staining revealed the luminal and exterior layers of the visceral muscle, and *mef2-GAL4* was specifically expressed in the exterior muscle layer of the intestine (Fig. 2C). In particular, the array of the nucleated muscle fibers were severely disturbed in *mef2* > *mst RNAi* flies (Fig. 2D). To dissect the critical visceral muscle layer for the intestinal dilation phenotype, we selectively knocked down *mst* expression using *mst* RNAi under the control of *how-GAL4*, 5053*A-GAL4* and *Myo1A-GAL4* which are specific for both the circular and the longitudinal visceral muscles, the longitudinal muscles only, and the enterocytes in the intestinal epithelium, respectively (Fig. 2E)^13,31–33^. In this experiment the circular muscle was not examined because we could not find a suitable GAL4 driver. Nonetheless, *how* > *mst RNAi* flies manifested the intestinal dilation phenotype as appeared in *mef2* > *mst RNAi* flies, but *Myo1A* > *mst RNAi* flies did not show any sign of the intestinal phenotype (Fig. 2F, second, third, and fifth panels of the upper column), confirming the previous notion that the visceral muscle is the critical locus for the intestinal dilation induced by *mst* RNAi expression. Intriguingly, the longitudinal muscle-specific knockdown of *mst* by *5053A-GAL4* driver (*5053* *A* > *mst RNAi*) elicited no considerable intestinal dilation, rather produced a mild thinner intestine compared to controls (Fig. 2F, fourth panel of the upper column). This longitudinal muscle-specific *mst* RNAi knockdown resulted in disruption only in the longitudinal muscle (Fig. 2F, fourth panel of the bottom column and inset), without significant defects in the circular muscle.

Consistent with these visceral muscle phenotypes, we observed that *how* > *mst RNAi* flies also exhibited severe defects in food intake and intestinal motility, and shortened life span similar to those of *mef2* > *mst RNAi* flies (Fig. S2F–H, respectively), but 5053 *A* > *mst RNAi* flies did not so. Together, these data suggest that the circular visceral muscle is likely important for *mst* depletion-induced intestinal defects or both of the nucleated visceral muscles are simultaneously important.

**Figure 3:**
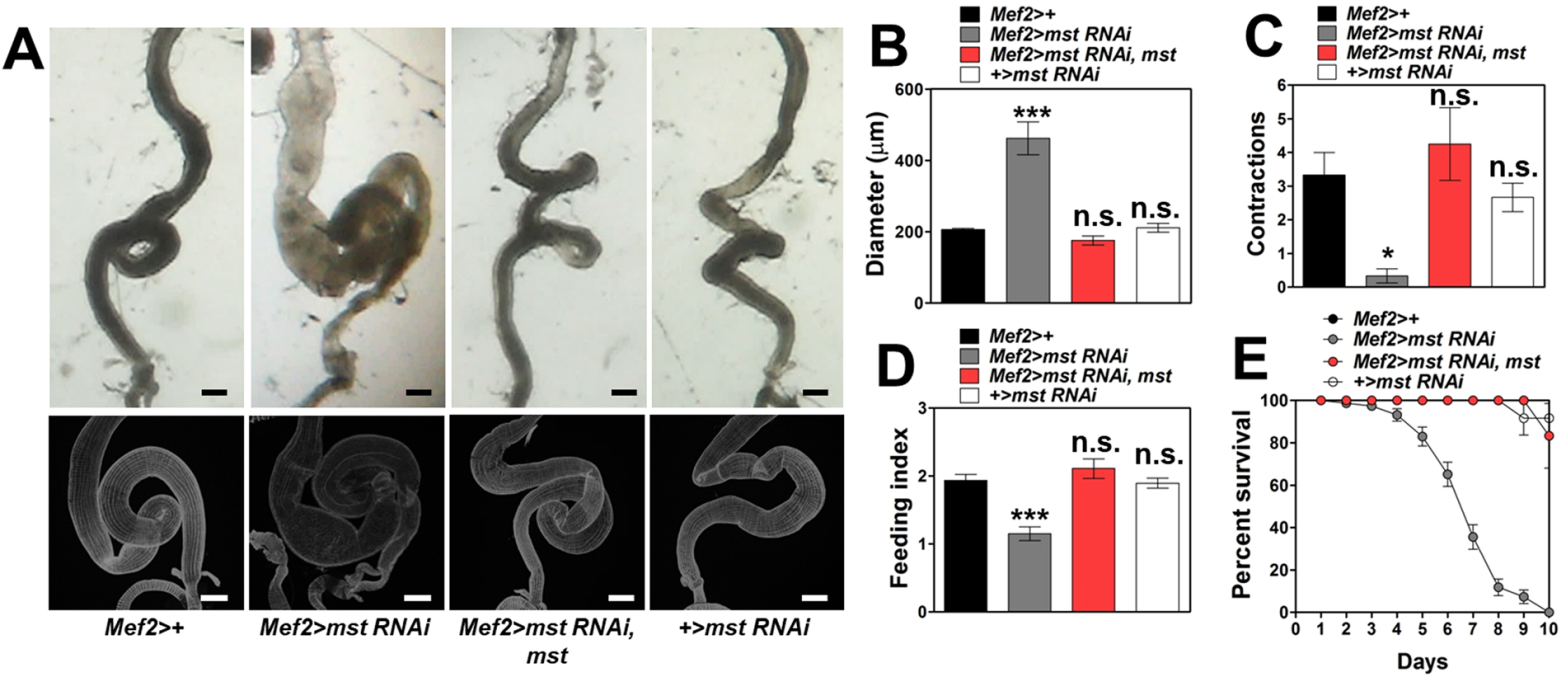
Genetic restoration of *mst* expression completely rescues all the *mst* RNAi-induced intestinal phenotypes. (**A**) Bright field and confocal images of the intestine bearing indicated genotypes stained with phalloidin. Scale bars: 200 μm. (**B**) Comparison of diameter of the intestine with indicated genotypes. N = 5–12. ***p < 0.0001; n.s. not significant by ANOVA Dunnett’s multiple comparison test. (**C**) Comparison of contraction frequencies of the intestine with indicated genotypes. N = 6–8. *p < 0.05; n.s. not siginificant by ANOVA Dunnett’s multiple comparison test. (**D**) Comparison of the level of food intake by the flies with indicated genotypes. N = 5. ***p < 0.0001; n.s. not siginificant by ANOVA Dunnett’s multiple comparison test. (**E**) Comparison of the survivorship of the flies with indicated genotypes. N = 6. Statistical significance was analyzed by Log-rank (Mantel-Cox) test. p < 0.0001.

Having shown that depletion of *mst* in the *Drosophila* visceral muscle elicits a series of intestinal phenotypes, we wondered whether genetic restoration of *mst* expression in *mef2* > *mst RNAi* flies would rescue the phenotypes. By combining *UAS-mst* transgene with *mef2* > *mst RNAi* flies, we observed that the dilated intestine of *mef2* > *mst RNAi* flies was completely normalized by the restoration of *mst* expression in the flies (Fig. 3A, third panel of the upper column, and 3B). Similarly, *mef2* > *mst RNAi* flies with restored *mst* expression exhibited normalized visceral muscle structures compared to *mef2* > *mst RNAi* and control flies (Fig. 3A, third panel of the bottom column). Likewise, restoration of *mst* expression in *mef2* > *mst RNAi* flies completely rescued the defects in intestinal motility, food intake, and life span (Fig. 3C–E, respectively). These data confirm the specific requirement of *mst* in mediating the intestinal phenotypes.

**Figure 4:**
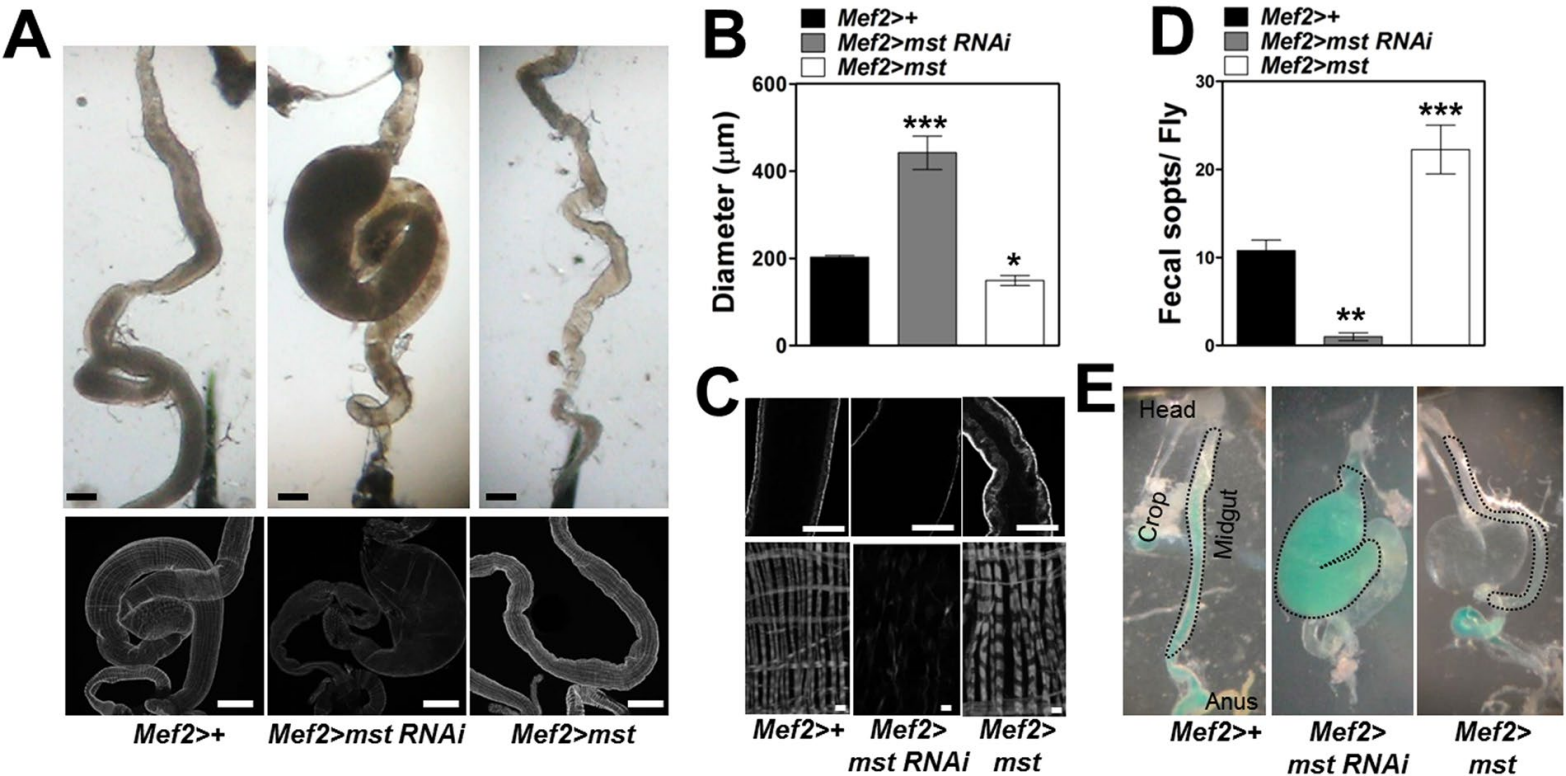
Exaggerated expression of *mst* produces intestine with reduced diameter, potentiated visceral muscle layer and thickened muscle fibers, and increased food transportation along the intestine and excretion. (**A**) Bright field and confocal images of the intestine bearing indicated genotypes stained with phalloidin. Scale bars: 200 μm. (**B**) Comparison of diameter of the intestine with indicated genotypes. N = 5–11. ***p < 0.0001; *p < 0.05 by ANOVA Dunnett’s multiple comparison test. (**C**) Horizontal section and high magnification of confocal images of the intestine bearing indicated genotypes stained with phalloidin. Scale bars: 100 and 5 μm. (**D**) Comparison of the fecal spots of the flies with indicated genotypes. N = 7. ***p < 0.0001; **p < 0.001 by ANOVA Dunnett’s multiple comparison test. (**E**) Bright field images of the intestine of the flies with indicated genotypes fed on colored food. Dotted lines denote the midgut. Scale bar: 200 μm.

Based on the observations that *mst* depletion elicited intestinal dilation along with weakened muscle layers, we wondered what would happen if we increase *mst* expression. Remarkably, overexpression of *mst* driven by *mef2-GAL4* produced intestine with potentiated muscle layer and thickened actin filaments, as opposed to *mst* depletion showing dilated intestine accompanying markedly weakened muscle layer and thin actin filaments (Fig. 4A–C, respectively). We hypothesized that the consolidated muscle structures by *mst* overexpression would cause increase in gut motility and excretion. Supporting this, we observed that the intestine with *mst* overexpression exhibited strikingly increased fecal spots, and increased gut contractions and movement of food down through intestinal tract, in contrast to *mst* depletion showing markedly decreased levels of fecal rate, gut contractions, and movement of food in the intestine (Fig. 4D and E; Movie S1).

**Figure 5:**
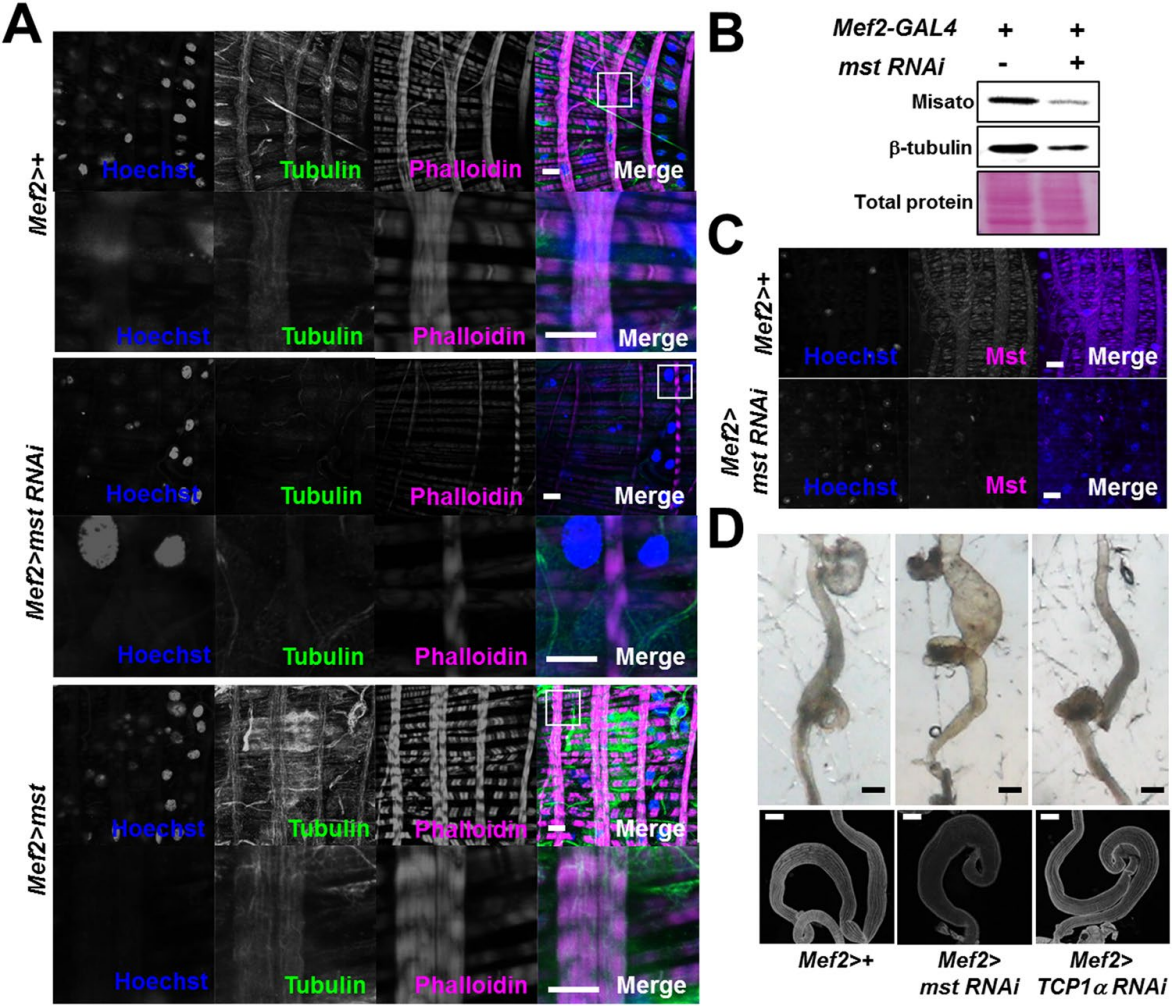
Visceral tubulin levels are altered by manipulation of *mst* expression. (**A**) Confocal images of visceral muscles with indicated genotypes stained with Hoechst, anti-tubulin antibody and phalloidin. Scale bars: 10 μm. White squares indicate the regions for the zoom-in images shown in the second rows of each genotype. (**B**) Immunoblot analysis of the level of tubulin and Mst expression using lysates from intestines of fifteen flies with indicated genotypes. These gel images were cropped from the original images with modified contrast and brightness for clarification. See Figure S3 for the raw gel images. (**C**) Confocal images of visceral muscles with indicated genotypes stained with Hoechst and anti-Mst antibody. Scale bars: 10 μm. (**D**) Bright field and confocal images of the intestine with indicated genotypes. Scale bars: 200 μm.

*Mst* was previously reported to function in microtubule formation interacting with TCP-1 tubulin chaperone complex^20^. To examine whether this is also the case in regulation of intestinal functions, we stained visceral muscles using an anti-tubulin antibody together with phalloidin and sought to compare the tubulin staining on visceral muscles of WT control, *mst*-depleted and *mst*-overexpressing flies, respectively. First of all, tubulin expression was detected along the muscle fibers and some unknown places in the visceral muscle (Fig. 5A, first row). In consistence with the previous report^20^, the tubulin staining in the visceral muscle was dramatically diminished upon the visceral depletion of *mst* compared to control (Fig. 5A, second row). Supporting this observation, decreased level of tubulin expression was detected from the intestine depleted for *mst* in comparison to that from control intestine (Figs 5B and S3). To ensure that Mst protein was successfully knocked down by the visceral expression of *mst RNAi* in these experiments, we immunoblotted and stained Mst protein in controls and *mst RNAi*-expressing intestine using a highly specific anti-Mst antibody. Both immunoblotting and histochemical staining experiments revealed marked reduction of Mst levels in the intestine (Figs 5B,C and S3). Intriguingly, overexpression of Mst in the visceral muscle increased tubulin expression (Fig. 5A, third row), indicating a tight linkage of Mst and tubulin expression/protein levels. Also the result that *mst*-knocked down visceral muscle showed reduction of tubulin expression led us to examine whether suppression of microtubule formation would elicit similar intestinal phenotypes as appeared in the *mst*-depleted intestine. However, we did not observe a significant intestinal defect by knockdown of TCP1α (Fig. 5D), the critical interacting partner of Mst in microtubule formation^20^. Intriguingly, Mst possesses two conserved tubulin-like motifs on the N-terminus^18^. To validate requirement of the motifs in regulation of the intestine, we generated a series of transgenic flies expressing truncated forms of Mst that lack the first tubulin-like motif (Mst Δ1), the myosin-like motif (Mst Δ2), or the second tubulin-like motif (Mst Δ3) (Fig. S4A)^18^. We expected that if these truncated proteins are functional, transgenic expression of them in *mst*-depleted intestine would rescue the defects induced by *mst-*depletion. Remarkably, the expression of Mst Δ1 and Mst Δ3 in *mst*-depleted flies did not rescue the defective life span in contrast to the expression of Mst Δ2 that completely rescued the defect similar to the level of WT *mst* expression (Fig. S4B). Together, these results suggest that the conserved tubulin-like motifs are critical for the function of Mst protein in the visceral muscle.

**Figure 6:**
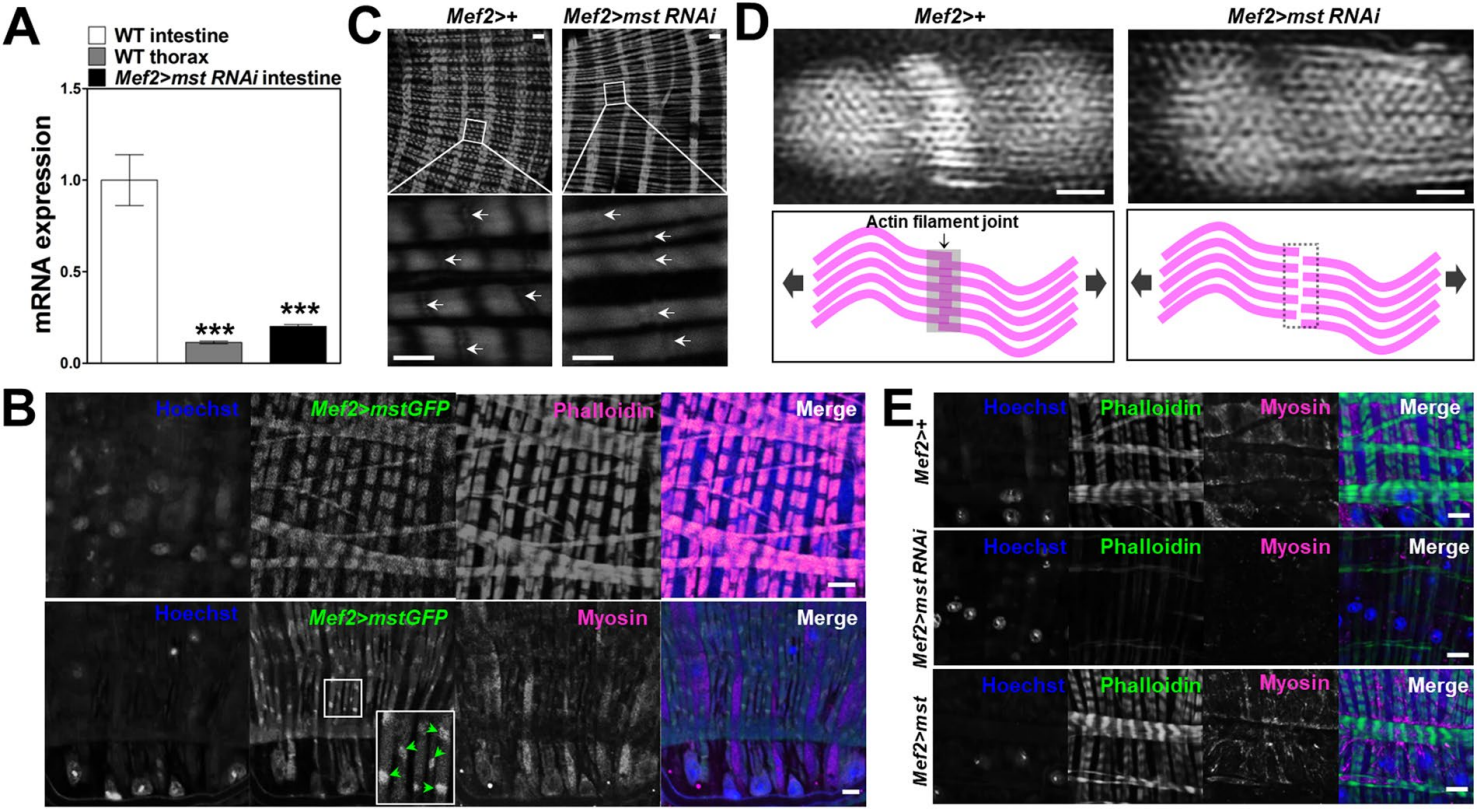
*Mst* is highly expressed in visceral actin filaments and involved in actin-myosin structures. (**A**) Comparison of the level of mRNA expression in the throax and intestine with indicated genotypes. N = 15. ***p = 0.0004 by ANOVA Dunnett’s multiple comparison test. (**B**) Confocal images of visceral muscle expressing *mef2* > *mstGFP* stained with Hoechst, phalloidin and anti-myosin antibody. Scale bars: 10 μm. White square and the inset illustrate the dense localization (arrowheads) of mstGFP in the segments of muscle fibers. (**C**) Confocal images of the visceral muscle with indicated genotypes stained with phalloidin. Scale bars: 10 and 5 μm. Note the arrows indicating the segments of muscle fibers that are lacked by *mst* depletion. (**D**) Super-resolution images of the actin filaments using OMX microscopy and a cartoon illustrating the joint of actin filaments in control and *mst*-depleted visceral muscles. Scale bars: 1 μm. (**E**) Confocal images of the visceral muscle with indicated genotypes stained with Hoechst, phalloidin and anti-myosin antibody. Scale bars: 10 μm.

Although *mst* expression was manipulated in whole muscle tissues of *Drosophila* in the above experiments using *mef2-GAL4* driver, the visceral muscle was specifically affected. We thus examined the visceral muscle-specificity of *mst* using quantitative real time PCR and found that *mst* showed higher expression in WT intestine (Fig. 6A, first bar), compared to the level of *mst* in the skeletal muscle (Fig. 6A, second bar). However, it is worthy to note that *mst* is indeed reported to be expressed at the highest level in the adult reproductive organs (www.flybase.org).

To take a close look into the expression of Mst protein in the visceral muscle, we expressed the GFP-fused Mst (Mst-GFP) protein using *mef2-GAL4* and sought to visualize its expression patterns in the muscle. We detected a characteristic stripe pattern of the Mst-GFP protein specifically co-localized to actin filaments in the visceral muscle cell (Fig. 6B, first panels) and also observed dense localization of Mst-GFP protein in the segments of visceral muscle fibers (Fig. 6B, second panels and inset). These observations were in agreement with the former staining result that revealed Mst expression patterns in the muscle fibers using an anti-Mst antibody (Fig. 5C). However, the overexpressed Mst-GFP protein was largely sequestered in the sarcoplasm of skeletal muscle cells (Fig. S5), suggesting the actin filament-associated role of Mst in the visceral muscle. We also observed that the flanking myosin filaments spanning the interval space of the stripes of actin filaments were not overlapped with the Mst-GFP protein (Fig. 6B, bottom panels), again suggesting the distinct interaction of Mst with visceral actin filaments. Supporting these staining results, we observed that *mst* depletion markedly weakened actin filaments compared to controls (Fig. 6C). Particularly, the segment structures of the muscle fibers were severely disrupted in *mst*-depleted visceral muscles (Fig. 6C, bottom panels). To investigate the role of Mst in the segment structure in a higher resolution, we used the OMX super-resolution microscopy and imaged the visceral muscle of control and *mst*-depleted intestine. We observed that the segments were densely crosslinked with actin filaments in controls (Fig. 6D, left panels), but the structure was missing in the *mst*-depleted visceral muscle (Fig. 6D, right panels). Based on this observation together with the specific localization of Mst protein in the segments of actin filaments, we speculated that Mst holds actin filaments to maintain visceral muscle structures (Fig. 6D, bottom panels). Also we observed that visceral actomyosin structures were diminished by *mst* depletion, but further consolidated by increased expression of *mst* (Fig. 6E). Together, these data suggest that *mst s*pecifically regulates visceral actomyosin structures through specific interaction with visceral actin filaments.

**Figure 7:**
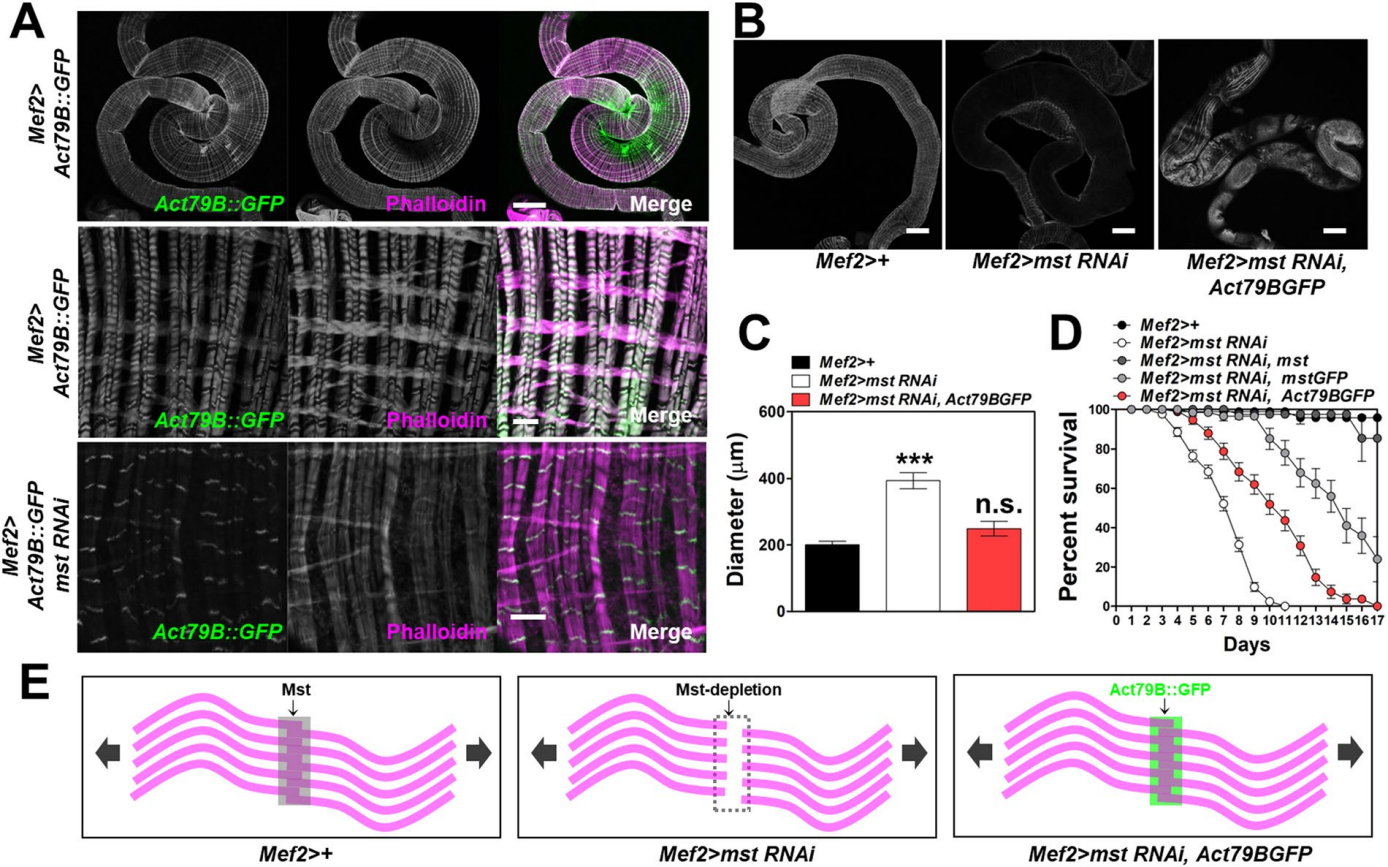
Exogenous expression of Act79B rescues the *mst* depletion-induced defects in the visceral muscle. (**A**) Confocal images of the intestine with indicated genotypes stained with phalloidin. Scale bars: 200 μm and 5 μm. (**B**) Confocal images of the intestine with indicated genotypes stained with phalloidin. Scale bars: 200 μm. (**C**) Comparison of the diameter of the intestine with indicated genotypes. N = 12–18. ***, p < 0.0005; n.s., not significant by ANOVA Dunnett’s multiple comparison test. (**D**) Comparison of the survivorship of the flies with indicated genotypes. N = 4–7. Statistical significance was analyzed by Log-rank (Mantel-Cox) test. p < 0.0001. (**E**) Schematic drawings of the structure of actin filaments. The joints highlighted by the colored or the dashed-line boxes with arrows to illustrate the putative dense bodies.

There are six actin members including *Act5c*, *Act42A*, *Act57B*, *Act79B*, *Act87E*, and *Act88F* in *Drosophila*. Their tissue specificities were reported as Act5C and Act42A, cytoplasmic; Act79B, leg and thorax; Act57B and Act87E, intersegmental muscles in larvae; Act88F, flight-muscle, although some members of these actin genes indeed share similar sequence identities, suggesting possible redundancy between them^34^. In search for a member of actins involved in the visceral muscle regulation, we sought to knockdown every member of actins and some actin-related proteins in the muscle by expressing their RNAi lines driven by *mef2-GAL4*. However, we found no RNAi lines against actin members and the related proteins producing intestinal phenotypes (Fig. S6 and Table S1). We would ascribe these results to the possible redundancy between the actin proteins or unsuccessful knockdown of the genes. Thus, instead, we sought to ectopically express each member of actins in the *mef2* > *mst RNAi* background to see whether the VM-like defects could be rescued. However, the expression of these actin members except for Act79B under the control of *mef2-GAL4* driver resulted in lethality probably due to toxicity caused by ectopic expression of these proteins in the muscle tissue (Table S1). Subcellular localization of Act79B driven by *mef2-GAL4* driver was specific to the actin filaments of the visceral muscle (Fig. 7A, first and second rows). Remarkably, this localization became dominant on the segments of the actin filaments upon *mst* depletion which caused impaired visceral actin filament structures (Fig. 7A, third row).

Although we did not provide experimental evidence, but by the morphology seen in the phalloidin staining, we suspected that the segment is the dense bodies (the counterpart of Z-lines in the skeletal muscle) that harbor some accessory proteins including actinin to crosslink actin filaments of adjacent actomyosin units (the counterpart of sarcomeres in the skeletal muscle)^35,36^. In consistence, *mef2-GAL4*-driven expression of Act79B::GFP significantly ameliorated the impairments of visceral actin fibers and intestinal dilation statistically indistinguishable from controls (Fig. 7B and C). Also the defective life span of the *mst*-depleted flies was dramatically rescued by the expression of Act79B::GFP in *mef2* > *mst RNAi* flies (Fig. 7D). These data led us to suggest that Act79B plays endogenous roles in the visceral muscle, and we speculate that the localization of Act79B to the segments may strengthen the structures of the dense bodies and consequently render them to hold actin filaments more tightly (Fig. 7E). Together, we suggest that Act79B genetically interacts and co-localizes with Mst in visceral actin filaments to maintain the intestinal muscle.

**Figure 8:**
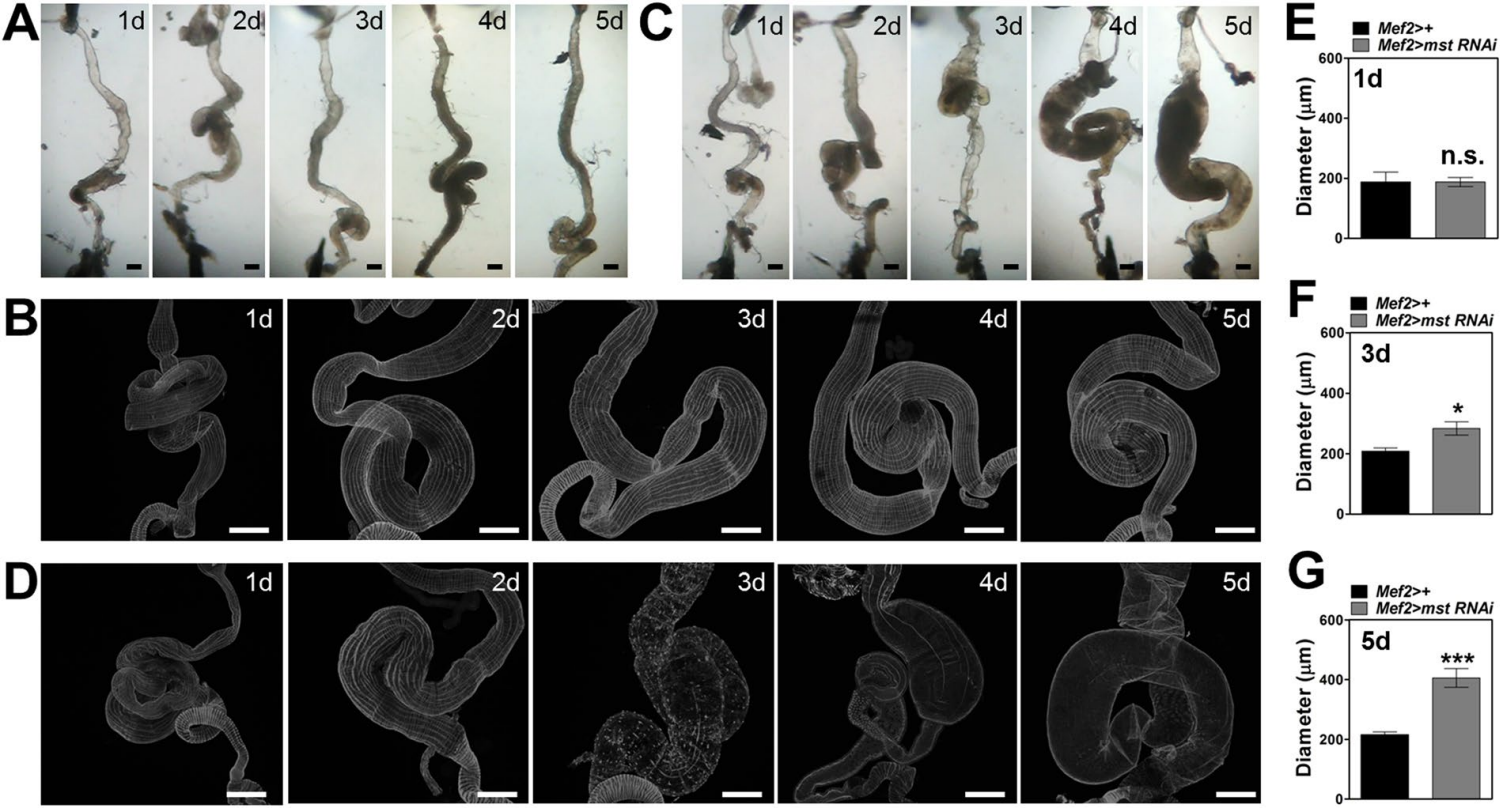
Visceral muscle degeneration by *mst* depletion occurs in an age-dependent manner. (**A**) Bright field images of the intestine of *mef2* *>* + flies on each day for 5 days. Scale bar: 200 μm. (**B**) Confocal images of the intestine stained with phalloidin of *mef2* > + flies on each day for 5 days. Scale bars: 200 μm. (**C**) Bright field images of the intestine of *mef2* > *mst RNAi* flies on each day for 5 days. Scale bars: 200 μm. (**D**) Confocal images of the intestine stained with phalloidin of *mef2* > *mst RNAi* flies on each day for 5 days. Scale bars: 200 μm. (**E–G**) Comparison of diameter of the intestine with indicated genotypes on 1, 3, 5 day. N = 4–9. ***p < 0.0001; *p < 0.05; n.s. not significant by unpaired t-test.

On the other hand, *mef2* > *mst RNAi* flies exhibited a tendency that aged flies showed more severely bloated phenotypes than young ones under visual inspection. To systematically monitor the time-dependent progress of the intestinal phenotypes, we examined morphological changes of the intestine and the visceral muscle from controls and *mef2* > *mst RNAi* flies for 5 days with 1 day-interval. In the beginning, the whole intestine and the visceral muscle from *mef2* > *mst RNAi* flies showed no significant morphological differences compared to controls (Fig. 8A–D, first panels; 8E). However, the visceral muscle of *mef2* > *mst RNAi* flies started to display abnormalities in muscle fibers from day 2 compared to controls (Fig. 8B and D, second panels). On day 3, the intestine showed dilation in a local area and the visceral muscle lost most muscle fibers with sporadic stumps as opposed to controls (Fig. 8A–D, third panels; 8F). Finally, the intestine of *mef2* > *mst RNAi* flies became completely bloated and the visceral muscle was degenerated on day 4 and 5 compared to controls (Fig. 8A–D, fourth and fifth panels; 8G). These observations indicate that there exists a degeneration mechanism that requires aging processes to propagate the visceral muscle degeneration induced by *mst* depletion.

**Figure 9:**
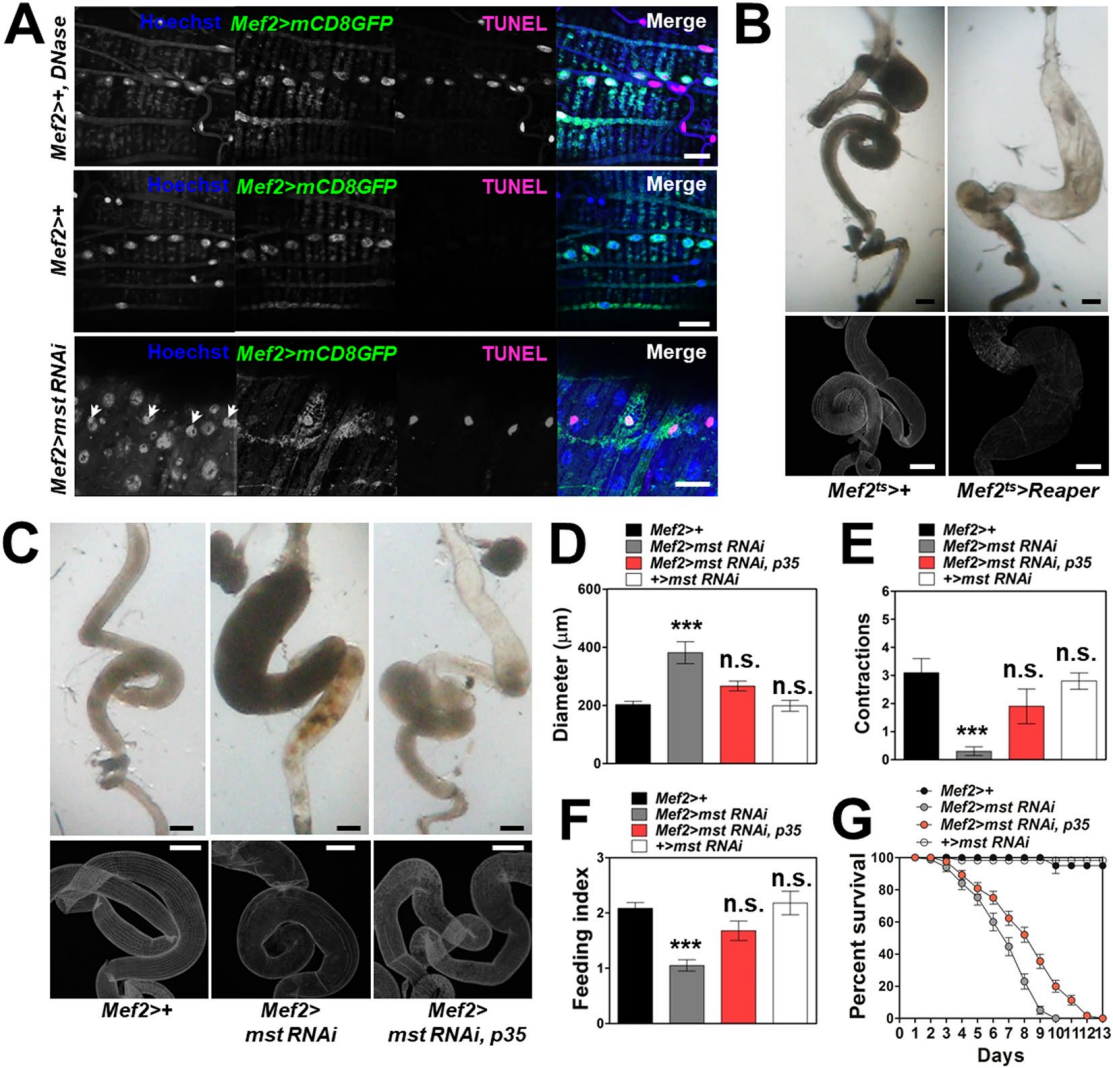
Visceral muscle apoptosis is increased along the progression of VM induced by *mst* depletion. (**A**) Confocal images of the visceral muscle with indicated genotypes expressing *mef2* > *mCD8GFP* stained with Hoechst and TUNEL apoptosis detecting method. Scale bars: 20 μm. (**B**) Bright field and confocal images of the intestine stained with phalloidin dissected from the flies with indicated genotypes reared at 30 °C. Scale bars: 200 μm. (**C**) Bright field and confocal images of the intestine with indicated genotypes stained with phalloidin. Scale bars: 200 μm. (**D**) Comparison of diameter of the intestine with indicated genotypes. N = 8–11. ***p < 0.0001; n.s. not significant by ANOVA Dunnett’s multiple comparison test. (**E**) Comparison of contraction frequencies of the intestine with indicated genotypes. N = 10. ***p = 0.0002; n.s. not significant by ANOVA Dunnett’s multiple comparison test. (**F**) Comparison of the level of food intake by the flies with indicated genotypes. N = 6. ***p = 0.0002; n.s. not significant by ANOVA Dunnett’s multiple comparison test. (**G**) Comparison of the survivorship of the flies with indicated genotypes. N = 5–6. Statistical significance was analyzed by Log-rank (Mantel-Cox) test. p < 0.0001.

Hinted from the previous report that siRNA-induced depletion of *MSTO1* caused fragmented mitochondrial morphology with apoptotic nuclear fragmentation leading to cell death in human cells^21^, we suspected that chronic apoptotic process occurred in the muscle is responsible for the visceral muscle degeneration. Remarkably, we observed severely fragmented mitochondria and increase of apoptotic responses in the visceral muscle from aged *mef2* > *mst RNAi* flies compared to young flies and controls (Figs S7, S8A, and 9A). To see whether artificial induction of apoptosis in the visceral muscle is sufficient to cause the intestinal dilation and muscle degeneration, we expressed Reaper, a pro-apoptotic component to induce apoptosis, in the visceral muscle using the temperature sensitive GAL80 combined with *mef2-GAL4* (*mef2* ^*ts*^ >+) which allows the GAL4-driven Reaper expression in the muscle only at 30 °C. Due to its lethal effect during development, we temporarily restricted the expression of Reaper only at adult stages by rearing *mef2* ^*ts*^ > *Reaper* flies at 18 °C during development and transferring the flies to 30 °C after eclosion. This temporal induction of muscle apoptosis resulted in intestinal dilation and visceral muscle degeneration similar to those by *mst* RNAi depletion in a condition where the induction persisted for long periods, but not within short time, suggesting that the visceral muscle apoptosis is a necessary factor for propagation of the *mst*-depletion-induced VM phenotypes (Figs S8B and 9B). To further substantiate the observation that the visceral apoptosis induced by *mst* depletion elicited the intestinal dilation and visceral muscle degeneration, we sought to rescue the intestinal phenotypes by suppressing apoptosis in the visceral muscle from *mef2* > *mst RNAi* flies by expressing p35 which has a strong anti-apoptotic activity. Consistent with our expectation, we observed that the disrupted visceral muscle structures and intestinal dilation by *mst* depletion was significantly rescued by the expression of p35 (Fig. 9C and D, respectively). Also other behavioral and physiological symptoms of *mef2* > *mst RNAi* flies, including gut contraction and food intake, were restored to up to the levels statistically indistinguishable from control flies (Fig. 9E and F, respectively), and the survivorship was markedly ameliorated by p35 expression in *mef2* > *mst RNAi* flies (Fig. 9G). Again, however, there were still some degree of remaining defects even by p35 expression and the VM phenotypes became further deteriorated upon aging (Fig. S8C). Based on these data, we suggest that the visceral muscle apoptosis poses downstream of the impaired visceral actomyosin structures induced by *mst* depletion.

## Discussion

In this study, we have shown that depletion of *mst* in the whole muscle tissues specifically impaired intestinal functions while skeletal muscles remained unaffected. The disrupted intestinal functions involve a spectrum of VM-like traits, such as degeneration of visceral muscle, dilation of intestinal tracts, decreased gut motility, reduced food intake and shortened life span. These pathological conditions induced by *mst* depletion seem to specifically correspond to the myopathic chronic intestinal pseudo-obstruction (myopathic CIPO) in human. There are two primary types of CIPO, one of which is neuropathic CIPO that results from disruption of the visceral nerves controlling the muscle contraction to generate peristalsis for mechanical digestion, and the other is called myopathic CIPO that is characterized by muscular abnormalities in the circular and longitudinal layers of visceral muscles^15^. The visceral myopathic conditions along with weakened peristalsis lead to intestinal obstruction without any mechanical obstructive processes that physically block the transportation of food along the gastrointestinal tract^37^. Although direct genetic and environmental causes for this disease have been obscure, damages on the smooth muscle of the gastrointestinal tract are a potential cause of the disease and some naturally-occurring mutations on the visceral muscle-specific actin genes are considered to be possible factors for the familiar form of myopathic CIPO^10,15,38–40^. In our experimental evidence, the myopathic CIPO phenotypes were similarly produced upon targeted-depletion of *mst* in the visceral muscle and were completely rescued by genetic restoration of *mst* expression, indicating that *mst* may specifically involves in the myopathic CIPO phenotypes. Supporting this, *mst* was expressed in the intestine and showed specific co-localization with the visceral actin filaments. Exaggerated expression of *mst* produced potentiated visceral muscle layers with thickened actin-myosin structures in contrast to *mst* depletion that attenuated the structures. As Mst is a highly conserved protein across various animal species (Fig. S9A), this Mst-mediated regulation of the visceral muscle may be conserved in metazoans.

Intriguingly, *mst* depletion specifically affected visceral actin filaments even though the depletion also occurred in the skeletal muscle that harbors abundant actin filament. This led us to hypothesized that there could be a type of visceral muscle-specific actins that correlate with Mst. Although actins are highly conserved and ubiquitous cytoskeleton proteins for all tissues, some actin isoforms, such as actin gamma 2 (ACTG2) and ACTA2, are reported to be specific for aortic and enteric smooth muscles^11,41^. In particular, a list of heterozygous missense variants in the *ACTG2* gene was identified by extensive exome sequencing on patients with familiar forms of VM^10,12^. As the control group was devoid of the *ACTG2* mutations, the mutations may be strongly correlated with familiar VM^10^. Our bioinformatic alignment and calculation on pathogenic proteins for intestinal diseases predicted that MSTO1 indeed shows a close relationship with ACTG2 (Fig. S9B). Interestingly, MSTO1 was linked to ACTG2 through filamin A (FLNA), an actin-binding protein that links actin filaments to the other cellular structures^42^, indicating that MSTO1 might interact with visceral actin filaments via an intermediate factor. In *Drosophila*, there are six genes encoding actin proteins: *Act5C*, *Act42A*, *Act57B*, *Act87E*, *Act88F*, and *Act79B* ^43^. In this study, our results support that Act79B likely interacts with Mst protein in visceral actin filaments to maintain the integrity of the visceral muscle.

## Electronic supplementary material

Supplementary Information

Movie S1
