# Supplementary material for: Misato underlies visceral myopathy in Drosophila
Source: Sci Rep. 2017 Dec 18;7:17700. doi: 10.1038/s41598-017-17961-3 (PMC5735100; doi:10.1038/s41598-017-17961-3)
Supplement: Supplementary file 1 — Supplementary Information [file 41598_2017_17961_MOESM1_ESM.pdf]

Misato underlies visceral myopathy in *Drosophila*

Soohong Min[1a,b], Woongchang Yoon[1,2a], Hyunho Cho[1,2] and Jongkyeong Chung[1, 2*]

[1]National Creative Research Initiatives Center for Energy Homeostasis Regulation, Institute of Molecular Biology and Genetics, and [2]School of Biological Sciences, Seoul National University, 1 Gwanak-Ro, Gwanak-Gu, Seoul 08826, Republic of Korea

[a]These authors equally contributed to this work.

[b]Present address: Harvard Medical School, Department of Cell Biology, 240 Longwood Avenue, Seeley-Mudd Building, Boston, MA 02115, USA

[*]Correspondence: jkc@snu.ac.kr (J.C.)

Supplementary Information Index

Supplementary Figures and Legends

Figure S1. Identification of *mst* RNAi from a feeding screen showing various impairments in the adult intestine.

Figure S2. Visceral muscle-restricted depletion of *mst* is sufficient to produce defects in food intake, gut contractions, and life span.

Figure S3. Depletion of *mst* using *mef2-GAL4* driver decreases the level of Tubulin and Mst protein in the intestine.

Figure S4. Tubulin-like motifs in Mst are required for normal function of *mst*.

Figure S5. Mst is localized in the sarcoplasm but not in the actin filament in skeletal muscle cells.

Figure S6. Depletion of actins and actin-related proteins in the visceral muscle does not elicit intestinal defects.

Figure S7. Depletion of *mst* results in fragmented mitochondria in the visceral muscle.

Figure S8. Visceral apoptosis is a necessary factor for propagation of VM.

Figure S9. Mst is conserved among animal species and interacts with diverse proteins.

Supplementary Movie Legend

Movie S1. The intestine with overexpressed *mst* exhibits increased contraction frequency.

Supplementary Table

Table S1. Intestinal phenotypes by RNAi depletion or transgenic expression of actin members and actin-related proteins driven by *mef2-GAL4* driver.

Supplementary Figures and Legends

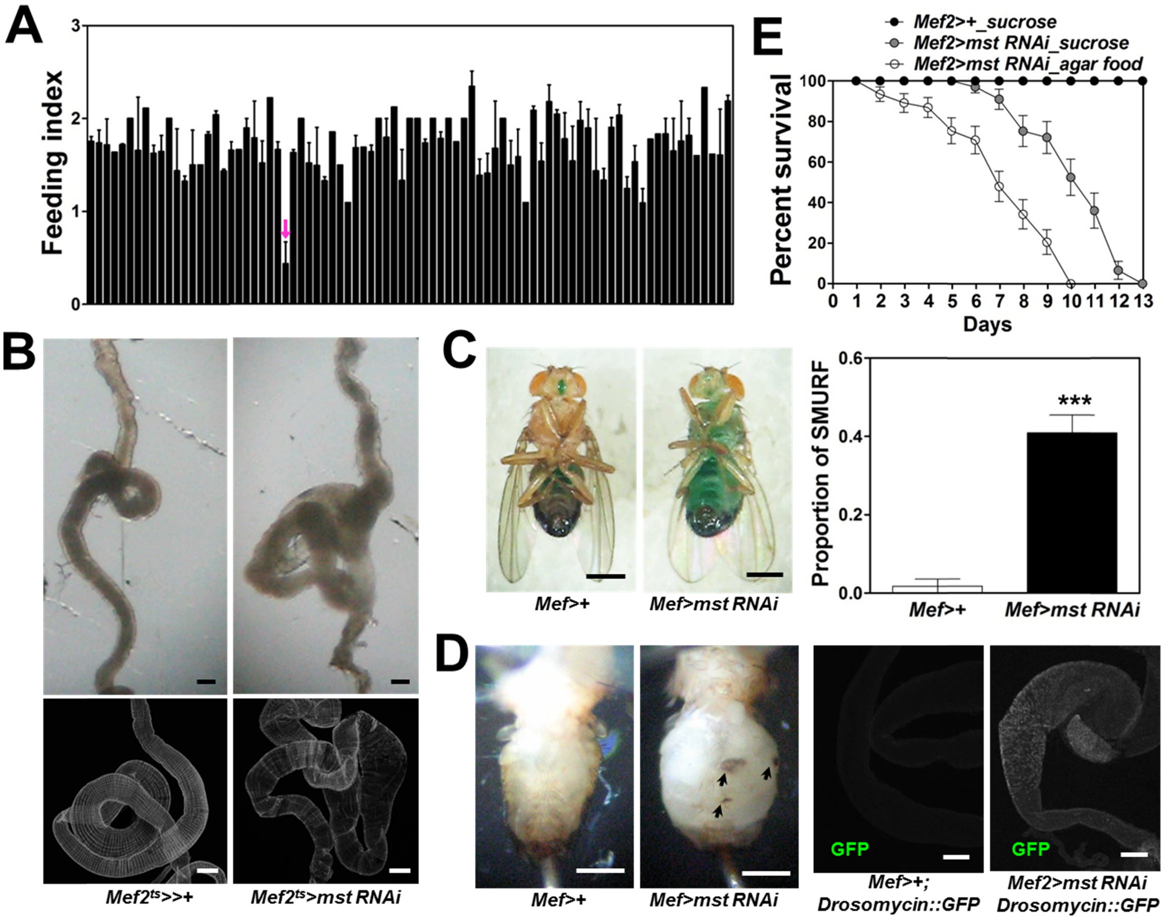

Figure S1. Identification of *mst* RNAi from a feeding screen showing various impairments in the adult intestine.

(A) Comparisons of the level of food intake by the flies harboring a copy of *UAS-RNAi* transgene driven by *Act5C-GAL4*, N=3. Arrow indicates *mst* RNAi. (B) Images of intestine dissected from the flies with indicated genotypes reared on 30°C. Scale bars: 200 µm. (C) Bright field images and comparison of proportion of the colored flies with indicated genotypes reared on green colored food and in their whole body. Scale bars: 0.5 mm. N=3. ***, $p<0.0005$ by unpaired t-test. (D) Bright field and confocal images of the abdomen and intestine with indicated genotypes. Arrows indicate the melanization by infection. Scale bars: 0.5 mm and 200 µm. (E) Comparison of the survivorship of the flies with indicated genotypes. N=3. Statistical significance was analyzed by Log-rank (Mantel-Cox) test. $p<0.0001$.

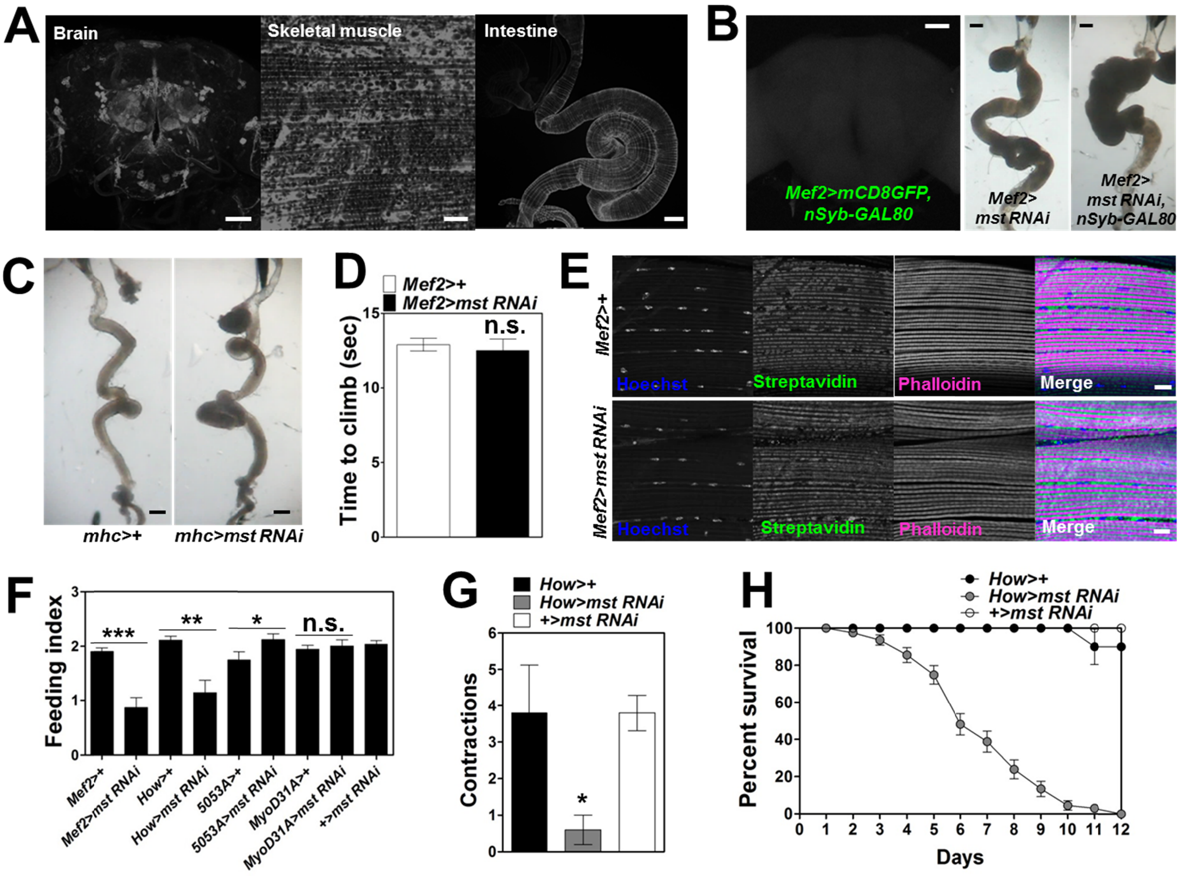

Figure S2. Visceral muscle-restricted depletion of *mst* is sufficient to produce defects in food intake, gut contractions, and life span.

(A) Confocal images of the indicated tissues expressing *mef2>mCD8GFP*. Scale bars: 50, 10 and 200 μm. (B) Confocal image of the brain and bright field images of the intestine with indicated genotypes. Scale bars: 50, 200 and 200 μm. (C) Bright field images of the intestine with indicated genotypes. Scale bars: 200 μm. (D) Comparison of the climbing ability of the flies with indicated genotypes. N=5-7. n.s. not significant by unpaired t-test. (E) Confocal images of the skeletal muscle in the thorax of the flies with indicated genotypes stained with Hoechst, streptavidin and phalloidin. Scale bars: 10 μm. (F) Comparison of the level of food intake by the flies with indicated genotypes. N=3-5. ***, $p<0.0005$; **, $p<0.005$; *, $p<0.05$; n.s. not significant by unpaired t-test. (G) Comparison of the contraction frequencies by the intestine with indicated genotypes. N=5. *, $p<0.05$ by ANOVA Dunnett's Multiple

comparison test. (H) Comparison of the survivorship of the flies with indicated genotypes. N=3-5. Statistical significance was analyzed by Log-rank (Mantel-Cox) test. $p<0.0001$.

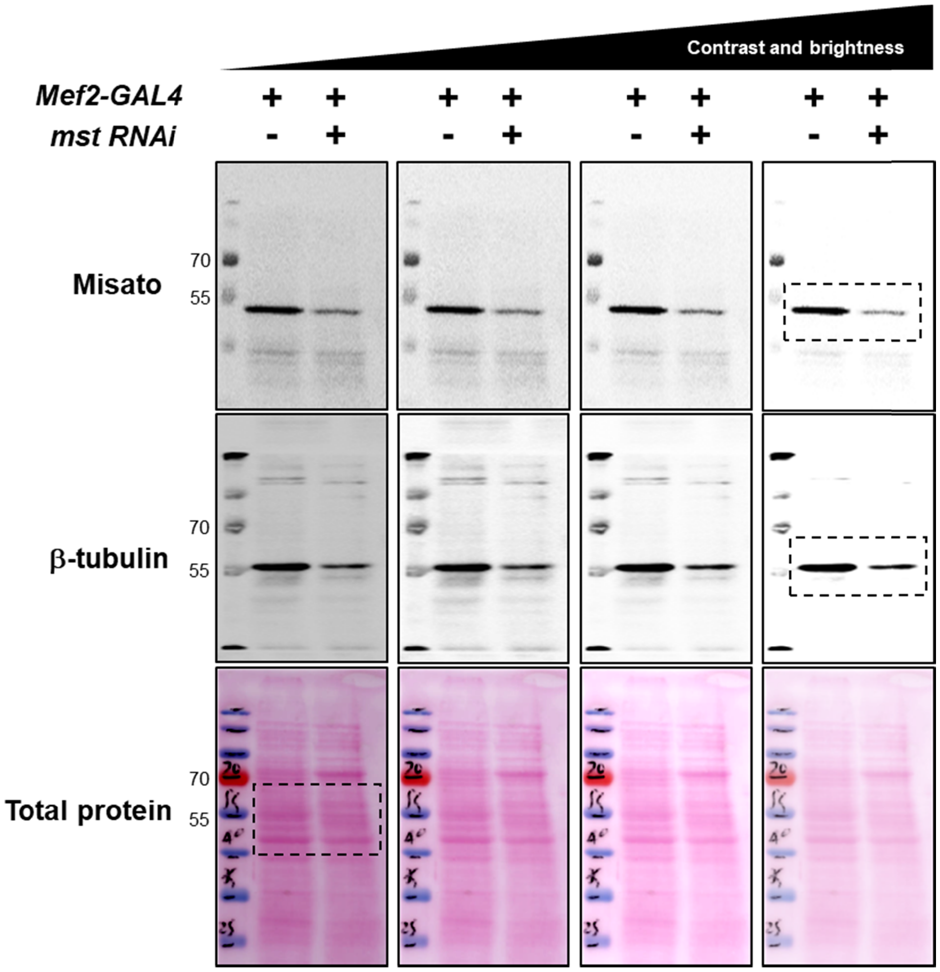

Figure S3. Depletion of *mst* using *mef2-GAL4* driver decreases the level of Tubulin and Mst protein in the intestine.

Immunoblot analysis of the level of Tubulin and Mst expression using lysates from intestines of fifteen flies with indicated genotypes. Multiple gel images by modifications of contrast and brightness are shown. Dashed boxes indicate the bands that are cropped to be presented in the main Figure 5B.

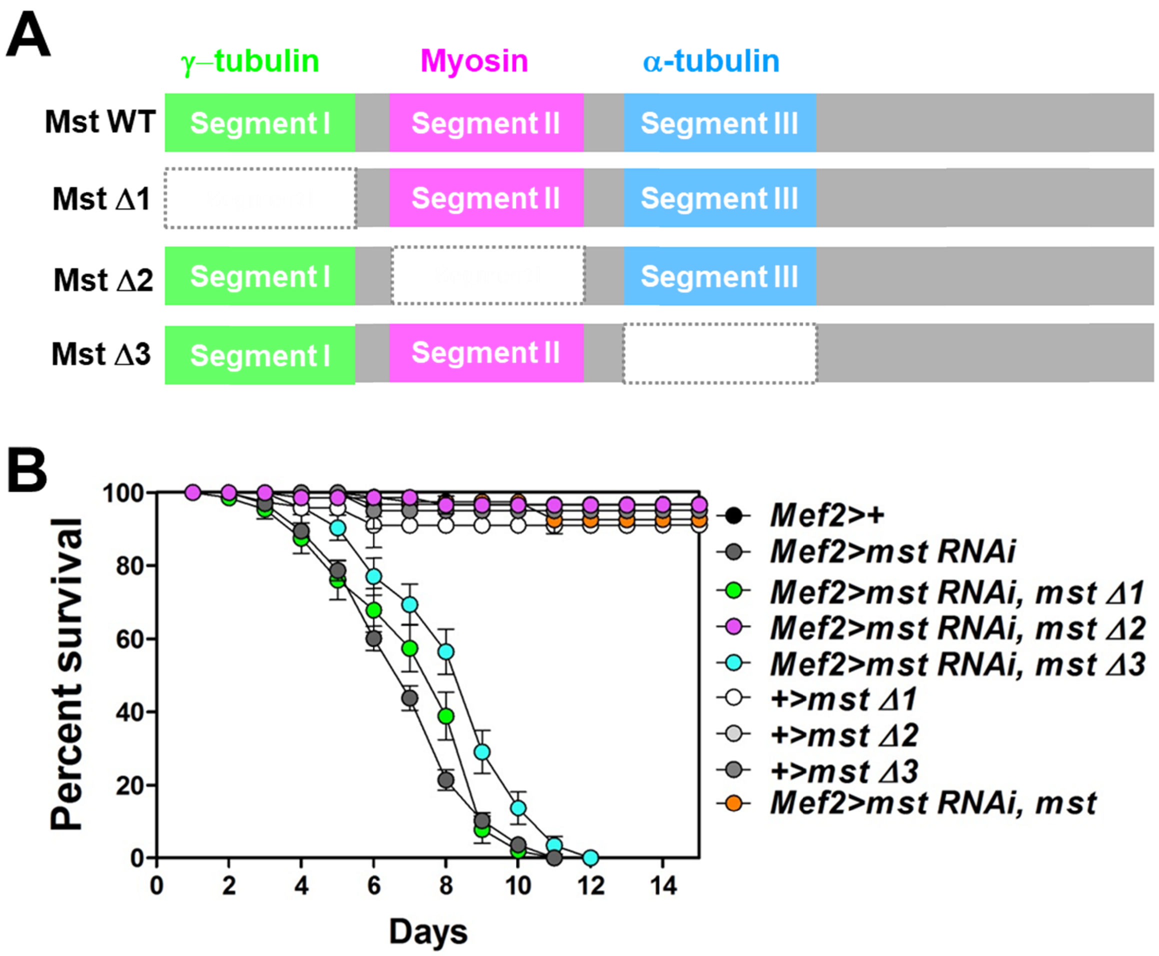

Figure S4. Tubulin-like motifs in Mst are required for normal function of *mst*.

(A) A cartoon that shows truncated forms of Mst protein harboring deletions of the conserved myosin and tubulin motifs. (B) Comparison of the survivorship of the flies with indicated genotypes. N=2-8. Statistical significance was analyzed by Log-rank (Mantel-Cox) test. $p<0.0001$.

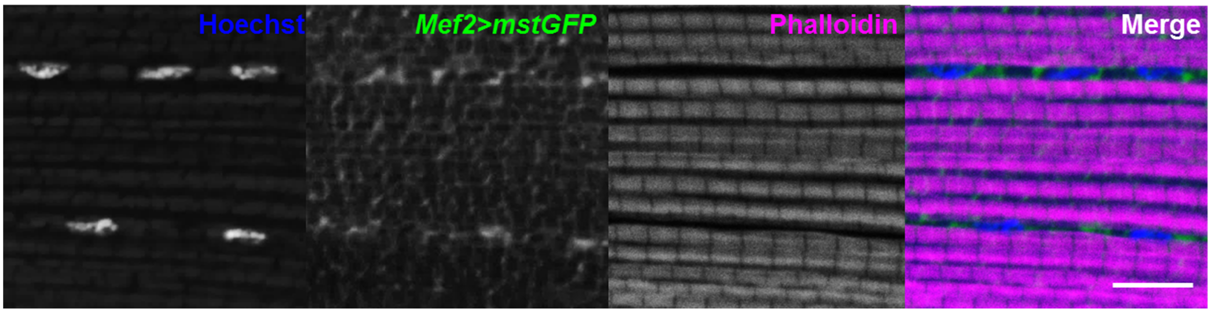

Figure S5. Mst is localized in the sarcoplasm but not in the actin filament in skeletal muscle cells.

Confocal images of the skeletal muscle expressing *mef2>mstGFP* stained with Hoechst and phalloidin. Note that the *mef2-GAL4* driven mstGFP sequestered in the sarcoplasm of muscle cells. Scale bar: 10 µm.

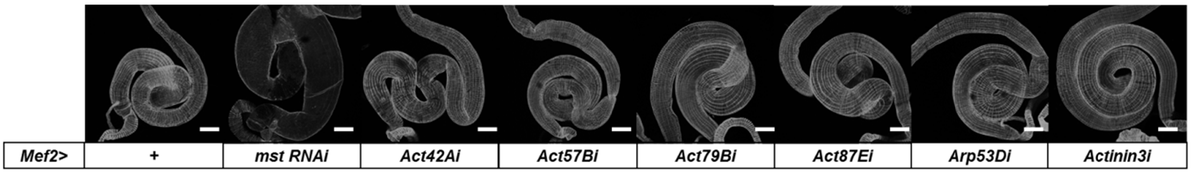

Figure S6. Depletion of actins and actin-related proteins in the visceral muscle does not elicit intestinal defects.

Confocal images of the intestine with indicated genotypes stained with phalloidin. Scale bars: 200 μm.

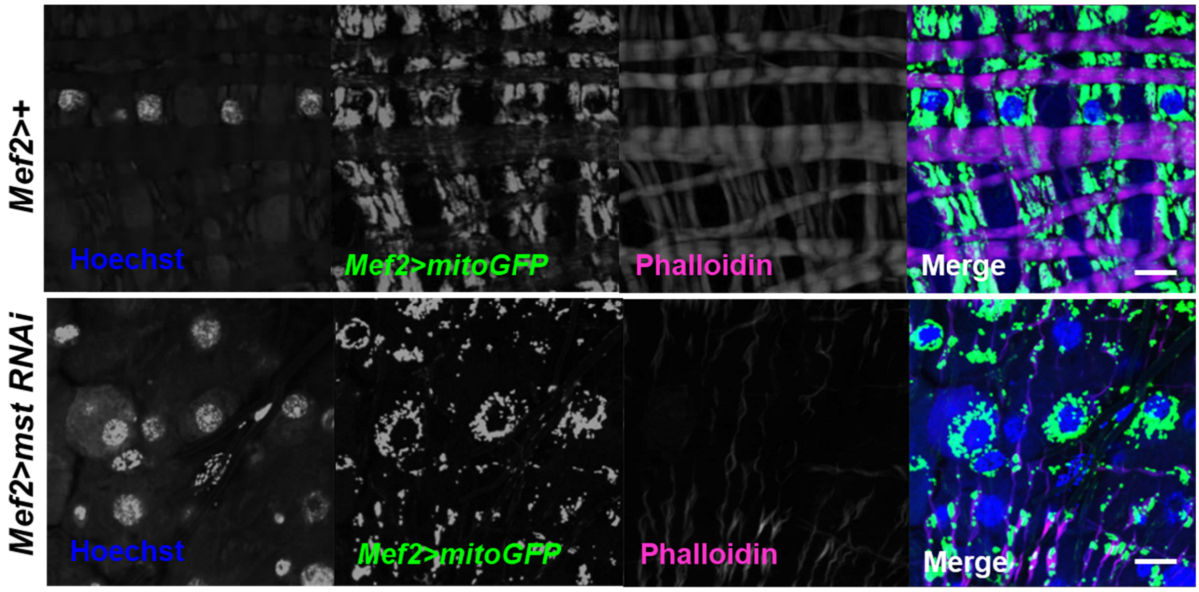

Figure S7. Depletion of *mst* results in fragmented mitochondria in the visceral muscle. Confocal images of visceral muscle with indicated genotypes stained for nucleus, *mef2>mitoGFP* and actin filaments. Scale bars: 10 µm.

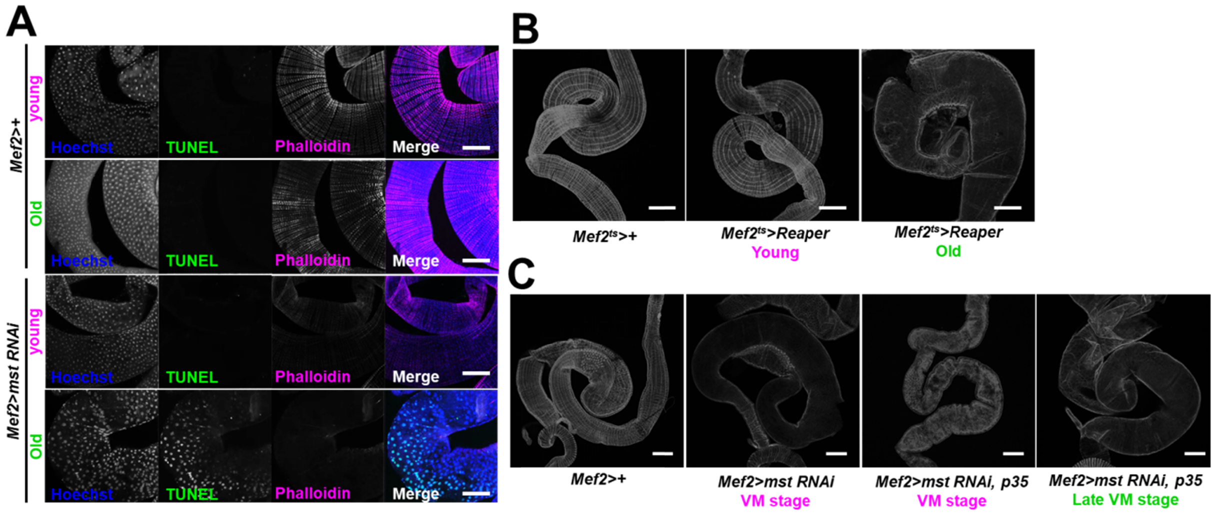

Figure S8. Visceral apoptosis is a necessary factor for propagation of VM.

(A) Confocal images of the intestine with indicated genotypes stained with Hoechst, TUNEL apoptosis detecting method and phalloidin. Young: ~ 3 day-old fly intestine; Old: ~ 7 day-old fly intestine. Scale bars: 100 μm. (B) Confocal images of the intestines of flies with indicated genotypes incubated at 30°C. The intestines were stained with phalloidin. Young: ~ 3 day-old fly intestine; Old: ~ 15 day-old fly intestine. Scale bars: 200 μm. (C) Confocal images of the intestine with indicated genotypes stained with phalloidin. VM stage: ~ 7 day-old fly intestine; Late VM stage: ~ 9 day-old fly intestine. Scale bars: 200 μm.

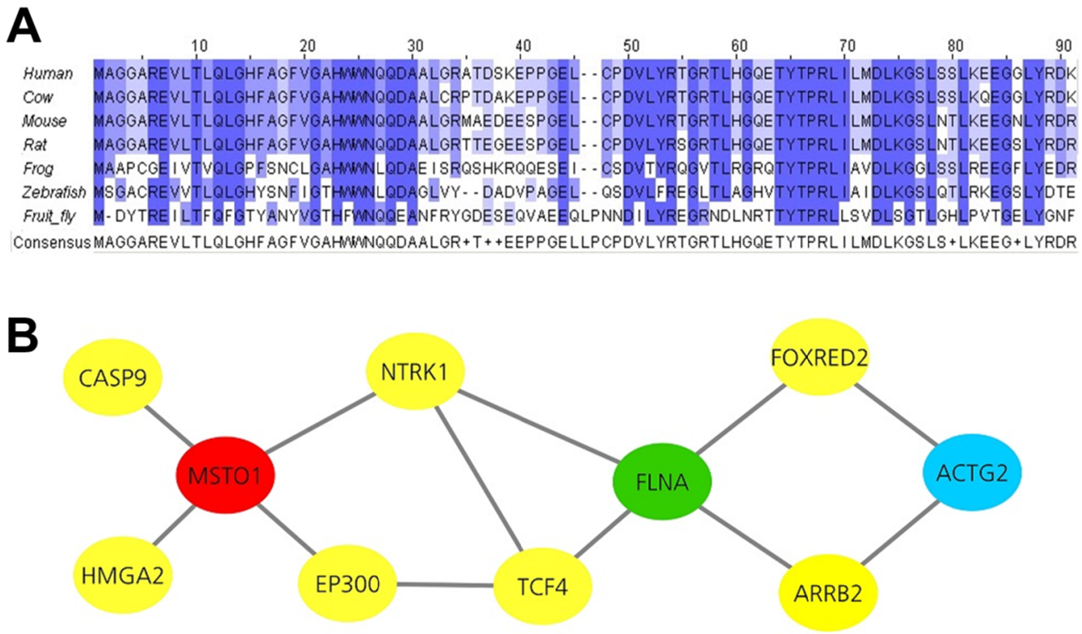

Figure S9. Mst is conserved among animal species and interacts with diverse proteins. (A) Multiple sequence alignment of the Mst orthologues from various species using Clustal Omega. (B) Gene-network analysis on human genes related to VM potentially interacting with MSTO1.

Supplementary Movie Legends

Movie S1. The intestine with overexpressed *mst* exhibits increased contraction frequency. Video recording of the contracting gut with indicated genotypes for 20 seconds. The clip speed was rescaled to 3 times faster.

Supplementary Table

Table S1. Intestinal phenotypes by RNAi depletion or transgenic expression of actin members and actin-related proteins driven by *mef2-GAL4* driver.

	Act5C	*Act42A*	*Act57B*	*Act79B*	*Act87E*	*Act88F*	*Arp53D**	*Actinin**	*Actinin3**	*Bent**
**UAS-RNAi X *Mef2-GAL4***	Lethal	Normal	Normal	Normal	Normal	Lethal	Normal	Lethal	Normal	Lethal
**UAS-Act::GFP X *Mef2-GAL4***	Lethal	Lethal	Lethal	expressed	Lethal	Lethal	N/A	N/A	N/A	N/A
**UAS-Act::GFP X *Mef2>mst RNAi***	Lethal	Lethal	Lethal	Rescued	Lethal	Lethal	N/A	N/A	N/A	N/A

*** These genes encode auxiliary proteins comprising actin filament structure.**
